# Structural insight into the cooperativity of spin crossover compounds

**DOI:** 10.1107/S2052520623005814

**Published:** 2023-08-11

**Authors:** H. Shahed, N. Sharma, M. Angst, J. Voigt, J. Perßon, P. Prakash, K. W. Törnroos, D. Chernyshov, H. Gildenast, M. Ohl, G. Saffarini, A. Grzechnik, K. Friese

**Affiliations:** a Jülich Centre for Neutron Science (JCNS-2) and Peter Grünberg Institute (PGI-4), Forschungszentrum Jülich GmbH, 52425 Jülich, Germany; b Institute of Crystallography, RWTH Aachen University, Jägerstr. 17-19, 52066 Aachen, Germany; c Department of Chemistry, University of Bergen, Allégaten 41, N-5007 Bergen, Norway; d Swiss–Norwegian Beamlines at the European Synchrotron Radiation Facility, 38000 Grenoble, France; e Institute of Inorganic Chemistry, RWTH Aachen University, 52074 Aachen, Germany; f Jülich Centre for Neutron Science (JCNS-1), Forschungszentrum Jülich, 52425 Jülich, Germany; g Department of Materials Science and Engineering, University of Tennessee, Knoxville, Tennessee 37996, USA; h Physics Department, An-Najah National University, Nablus, Palestine; i Jülich Centre for Neutron Science (JCNS-4), Forschungszentrum Jülich GmbH, 52425 Jülich, Germany; University of Erlangen-Nürnberg, Germany

**Keywords:** spin crossover,, radiation damage, thermal cyclic, entropy, intermolecular interactions

## Abstract

Spin-crossover (SCO) compounds are promising materials for a wide variety of industrial applications. However, the fundamental understanding of their nature of transition and its effect on the physical properties are still being fervently explored; the microscopic knowledge of their transition is essential for tailoring their properties. Here an attempt is made to correlate the changes in macroscopic physical properties with microscopic structural changes in the orthorhombic and monoclinic polymorphs of the SCO compound Fe(PM-Bia)_2_(NCS)_2_ (PM = *N*-2′-pyridyl­methyl­ene and Bia = 4-amino­biphenyl) by employing single-crystal X-ray diffraction, magnetization and DSC measurements. The dependence of macroscopic properties on cooperativity, highlighting the role of hydrogen bonding, π–π and van der Waals interactions is discussed. Values of entropy, enthalpy and cooperativity are calculated numerically based on the Slichter–Drickamer model. The particle size dependence of the magnetic properties is probed along with the thermal exchange and the kinetic behavior of the two polymorphs based on the dependence of magnetization on temperature scan rate and a theoretical model is proposed for the calculation of the non-equilibrium spin-phase fraction. Also a scan-rate-dependent two-step behavior observed for the orthorhombic polymorph, which is absent for the monoclinic polymorph, is reported. Moreover, it is found that the radiation dose from synchrotron radiation affects the spin-crossover process and shifts the transition region to lower temperatures, implying that the spin crossover can be tuned with radiation damage.

## Abstract 2

Macroscopic physical properties are correlated with microscopic structural changes in the orthorhombic and monoclinic polymorphs of the spin crossover compound [Fe(PM-BiA)_2_(NCS)_2_] by employing single crystal X-ray diffraction, magnetization and DSC measurements. Focus is given on the kinetic behavior and a theoretical model is proposed for the calculation of the non-equilibrium spin-phase fraction.

## 1. Introduction

Some of the 3-*d* transition metal complexes show a switching between two or more spins states of the central cation induced by a perturbation of external conditions (*T*, *P*, *h*ν) (Kahn & Martinez, 1998 ▸; Gütlich *et al.*, 2007 ▸; Levchenko *et al.*, 2014 ▸). This phenomenon is called spin crossover and was first observed more than 90 years ago (Cambi & Cagnasso, 1931 ▸; Cambi & Szegö, 1933 ▸). For Fe^2+^ in a (nearly) octahedral ligand field of a certain strength, a low-spin (LS, singlet *t*_2*g*_^6^) state can be switched to a high-spin (HS, quintet *t*_2*g*_^4^ *e*_g_^2^) state by heating; here, the entropy associated with a change in electronic multiplicities and vibrational frequencies is a leading driving force (Grandjean *et al.*, 1989 ▸). An important parameter is the temperature of spin state equilibrium *T*_½_ = Δ*H*/Δ*S*, where Δ*H* and Δ*S* are enthalpy and entropy changes, respectively, linked to the change of the spin state. A difference in molecular volumes (LS state shows shorter Fe to ligand distances compared to HS state) makes LS states favorable under external pressure which is, therefore, yet another parameter controlling the spin states (von Ranke, 2017 ▸; Gütlich *et al.*, 2007 ▸). An external perturbation may induce a gradual conversion of spin states (a crossover) or an abrupt switch with significant hysteresis (a first-order spin state transition). Strength and range of intermolecular interactions are believed to define the shape of the transition curve (*i.e.* the fraction of complexes in HS state plotted as a function of external perturbation, *e.g.* temperature or pressure) (Gütlich *et al.*, 2013 ▸).

The change in spin state is associated with a change in magnetization, unit-cell volume and color; this is why materials undergoing a spin crossover transition have attracted wide interest for many potential applications, *e.g.* information storage, optical devices and displays (Létard *et al.*, 2003 ▸; Ksenofontov *et al.*, 2004 ▸; Tuan, 2012 ▸; Brooker, 2015 ▸; Kahn *et al.*, 1992 ▸; Kahn & Martinez, 1998 ▸). Recently, spin-crossover materials have been discussed as solid-state materials for caloric applications (Vallone *et al.*, 2019 ▸; Sandeman, 2016 ▸; Romanini *et al.*, 2021 ▸; von Ranke, 2017 ▸; Reis, 2020 ▸), as the spin crossover transition is susceptible to pressure (*P*). For potential barocaloric applications, a requirement of the spin crossover material is a strong dependence of the temperature of spin state equilibrium on pressure; according to Sandeman (2016 ▸), the expected caloric effect is proportional to d*T*_½_/d*P*.

**Figure 1:**
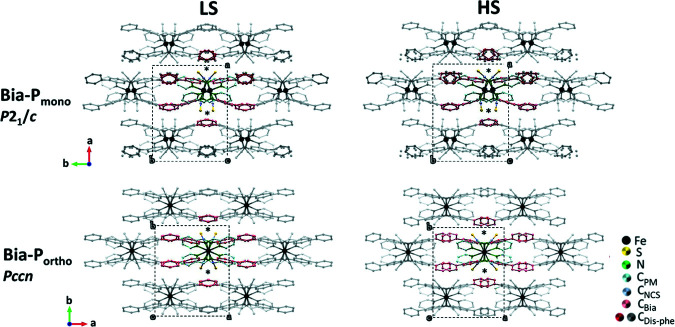
View of the crystal packing of [Fe(PM–Bia)_2_(NCS)_2_] complex in the LS state (left) and HS state (right). Projection along the *c* direction for both polymorphs **Bia-P_mono_** (top) and **Bia-P_ortho_** (bottom). Hydrogen atoms are omitted for clarity. The marker * highlights the difference between the thio­cyanate branch and the phenyl rings in the LS and HS, respectively.

An attractive candidate for barocaloric research is the [Fe(PM-Bia)_2_(NCS)_2_] complex (Figs. 1 ▸, 2 ▸ and S1), where PM is *N*-2′-pyridyl­methyl­ene and Bia is 4-amino­biphenyl (Sandeman, 2016 ▸); this is probably the most studied spin crossover material (Létard *et al.*, 1998 ▸). The large volume of reported data on structure and properties helps us to identify the genuine spin-crossover response not affected by a particular experimental or sample preparation protocol. [Fe(PM-Bia)_2_(NCS)_2_] can crystallize in two different polymorphs, an orthorhombic one with space group *Pccn* (denoted as **Bia-P_ortho_** hereinafter) and a monoclinic one with space group *P*2_1_/*c* (denoted as **Bia-P_mono_** hereinafter). There is a report on a third polymorph stable at high pressure, an intermediate state (Rotaru *et al.*, 2009 ▸), but available structural information is not conclusive. Similar intermediate states induced by variable scan rates have also been reported for other SCO compounds (Ridier *et al.*, 2018 ▸; Chakraborty *et al.*, 2012 ▸; Fujinami *et al.*, 2015 ▸; Li *et al.*, 2022 ▸).

The magnetic behavior of the two polymorphs is different: whereas **Bia-P_ortho_** shows a very abrupt SCO transition in a narrow temperature range of about 1 K at ∼175 K with a thermal hysteresis of 5 K (Rodríguez-Velamazán *et al.*, 2007 ▸; Létard *et al.*, 1998 ▸, 1999 ▸, 2003 ▸; Ksenofontov *et al.*, 1998 ▸), **Bia-P_mono_** shows a gradual spin crossover with *T*_½_ at approximately 210 K, which stretches over a large temperature range from 150 K to 250 K (Guionneau *et al.*, 1999 ▸, 2001 ▸; Létard *et al.*, 1999 ▸).

In this study, we re-examine single-crystal structures of both polymorphs as a function of temperature, and probe rate-dependent magnetization. We also present synchrotron diffraction data collected in a cyclic mode with a fine temperature sampling uncovering the full temperature evolution of various intra- and intermolecular contacts, as well as lattice deformations and atomic displacement parameters (ADPs). Another goal of our study is to see whether scan rate-dependent measurements lead to an intermediate state or show any sizeable kinetic hysteresis for **Bia-P_mono_** and **Bia-P_ortho_**. In addition, synchrotron powder diffraction data uncover irreversible lattice deformation which can be attributed to radiation damage. The above results of temperature-dependent experiments are parameterized with a phenomenological thermodynamic model.

## 2. Experimental

### 2.1. Sample preparation

The title compound **Bia-P_mono_** was prepared according to reported chemical synthesis procedures (Létard *et al.*, 1998 ▸). Purity and phase identity were confirmed with elemental analysis and powder X-ray diffraction (Fig. S2). Single crystals of **Bia-P_mono_** were prepared by layering a solution of [Fe(NCS)_2_(py)_4_] (12.2 mg, 0.025 mmol) in methanol (1 ml) with a solution of the ligand Bia-PM (12.9 mg, 0.05 mmol) in di­ethyl ether (1 ml) in an inert N_2_ atmosphere. Between the two layers, a layer of the 1:1 mixed solvents (1 ml) was placed to slow the reaction resulting in the formation of single crystals after one week.

Single crystals of **Bia-P_ortho_** were prepared by placing [Fe(NCS)_2_(py)_4_] (48.8 mg, 0.1 mmol) and the ligand Bia-PM (51.6 mg, 0.2 mmol) in separate sides of an H-tube and slowly adding methanol until the solvent connected the two solids. Single crystals grew after two weeks.

### 2.2. Magnetic susceptibility measurements

The magnetic susceptibility measurements were performed on a bunch of single crystals using the RSO option of a Quantum Design magnetic property measurements system (MPMS XL) applying a constant field of μ_0_ *H* = 2 T and 50 mT for **Bia-P_ortho_** and **Bia-P_mono_**, respectively.^1^ For polycrystalline samples, the measurements were performed using the VSM (vibrating sample magnetometer) option of the Physical Property Measurement System Dynacool by Quantum Design, with a constant magnetic field of μ_0_ *H* = 50 mT. 

The temperature dependence of the zero-field-cooled (ZFC), the field-cooled cooling (FCC) and the field-cooled warming (FCW) magnetization were measured in the temperature range 5 K < *T* < 350 K with different temperature rates of 10, 8, 5, 2, 1, 0.5 and 0.2 K min^−1^ for both polymorphs. All data were collected in sweep mode. For both polymorphs, the measurements were performed by cycling several times (four) at a rate of 0.2 K min^−1^. Both diamagnetic and paramagnetic corrections were applied for the contribution of the sample holder and the sample itself.

### 2.3. Differential scanning calorimetry

Calorimetric measurements were performed on samples sealed in aluminium pan TA instruments DSC Q2000 calorimeter. The measurements were performed with a scan rate of 10 K min^−1^. A heating/cooling cycle was applied in the temperature range of 313 K to 143 K to 313 K with an equilibration time of 1 min at 313 K and at 143 K.

### 2.4. Synchrotron powder X-ray diffraction for **Bia-P_mono_**

High-resolution (3.7 mdeg in 2θ) powder diffraction data were collected at the material science beamline X04SA-MS (Willmott *et al.*, 2013 ▸) at the Swiss Light Source, PSI, Switzerland, using a wavelength of 0.708 Å. A capillary of 0.3 mm diameter was filled with the sample and an OXFORD cryojet was used to cool the sample. A MYTHEN II detector (Bergamaschi *et al.*, 2010 ▸) by DECTRIS was used. Data were collected in the following way: first the sample was cooled down at a rate of 6 K min^−1^ to 100 K, then the data were measured while warming up (at a rate of 6 K min^−1^) to 300 K in 5 K temperature steps. In the second run data were measured while cooling down (2.5 K min^−1^) from 300 K to 100 K in 5 K temperature steps. Finally, data were remeasured while warming up (2.5 K min^−1^) to 300 K. Unit-cell parameters were obtained from Le Bail refinements (Le Bail & Fourquet, 1992 ▸) using the program *Jana2006* (Petříček *et al.*, 2014 ▸). The refined parameters included unit-cell parameters, background parameters (ten polynomial coefficient), pseudo-Voigt profile parameters (GW and LY), zero shift and anisotropy stain broadening parameters (St400, St103, St004). All parameters were refined in alternating cycles. Berar’s correction (Bérar & Baldinozzi, 1993 ▸) was applied in order to obtain a realistic standard deviation.

### 2.5. Synchrotron single-crystal X-ray diffraction

High-resolution single-crystal X-ray diffraction data were collected at the Swiss Norwegian Beamline BM01A at the ESRF in Grenoble, France, using a PILATUS 2M detector (Dyadkin *et al.*, 2016 ▸). An Oxford Crystream 700+ was employed for cooling. The sample to detector distance and the detector parameters were calibrated using an alum single crystal standard.

The measurements were carried out using a wavelength of 0.630 (5) Å for **Bia-P_mono_** and the data were collected while cooling down from 270 K to 93 K with a temperature step of 3 K. Temperature was changed at a rate of 6 K min^−1^ with a waiting time of 2 min per step.

Data on **Bia-P_ortho_** were measured using a wavelength of 0.650 (5) Å. Data were collected in the following way: first, the sample was heated at a rate of 6 K min^−1^ to 350 K, the sample was maintained at 350 K for 1 min for thermal equilibration, then the data were measured on cooling (at a rate of 6 K min^−1^ with a waiting time of 3 min per step) from 350 K to 85 K in 5 K temperature steps. In the second run, data were measured on warming from 85 K to 350 K in 5 K temperature steps.^2^ In the third run, the data were re-measured on cooling from 300 K to 200 K in 50 K temperature steps and then with smaller temperature steps of 1 K in the transition range from 190 K to 165 K. Finally, data were re-measured on warming from 165 K to 190 K in 1 K temperature steps. 

Data processing was performed using *SNBL ToolBox* software [a Swiss army knife for Pilatus data (Dyadkin *et al.*, 2016 ▸)] which was developed at the beamline. The integration of the intensities and subsequent data reduction was performed using the *CrysAlis Pro* program (Rigaku Oxford Diffraction, 2018 ▸). The structure was solved via direct methods using the *SHELXT* software (Sheldrick, 2015*b* ▸). Sequential structure refinements were performed using *SHELXL* (Sheldrick, 2015*a* ▸). Hydrogen atoms were introduced and their positions were fixed using the appropriate geometrical constraints. All non-hydrogen atoms were refined anisotropically. The disordered phenyl group was treated as a rigid group with C—C and C—H bond distances set to 1.39 Å and 0.93 Å, respectively. Overall agreement factors for the refinements assuming the same sinθ/λ limits are significantly better than the ones reported in the literature (Létard *et al.*, 1998 ▸; Marchivie *et al.*, 2003 ▸) for earlier refinements (Table S1).

## 3. Results

### 3.1. Magnetic properties

The temperature-dependent magnetization measurement for the monoclinic polymorph **Bia-P_mono_** shows a gradual spin transition ranging from 250 K to 150 K covering a wide transition region; the orthorhombic polymorph **Bia-P_ortho_** shows an abrupt transition with *T*_½_↑ = 173 K and *T*_½_↓ = 167 K (thermal hysteresis of 6 K). Both results are in good agreement with the literature (Létard *et al.*, 2003 ▸, 1998 ▸; Ksenofontov *et al.*, 1998 ▸; Guionneau *et al.*, 1999 ▸). This confirms that our material meets the standards set by previous groups in terms of purity and properties.

Measurements on single crystals of both polymorphs using different scan rates (Figs. 3 ▸, views *a*1 and *a*2) show a negligible scan rate dependence of the thermal hysteresis with nearly constant values of 0.8 K for **Bia-P_mono_** and 6.5 K for **Bia-P_ortho_** (Fig. 3 ▸, views *b*1 and *b*2). On the other hand, measurements on the polycrystalline samples of both polymorphs show that, while the shape of the transition curve remains similar (Fig. 3 ▸, views *a*3 and *a*4) with increasing the scan rate from 0.2 K min^−1^ to 10 K min^−1^, the width of the hysteresis curve undergoes a monotonic increase from 0.6→12.3 K (Δ*T* = 11.7 K) for **Bia-P_mono_** and from 3.2→17.9 K (Δ*T* = 14.7 K) for **Bia-P_ortho_** (Fig. 3 ▸, views *b*3 and *b*4). Furthermore, the measurement on the polycrystalline sample of **Bia-P_ortho_** reveals a two-step transition with an intermediate state observed only during the warming cycles (see below) (Fig. 3 ▸, view *a*4). The two-step character becomes more pronounced with lower scan rates. As previous investigations of the magnetic properties were focused on fast scan rates, this behavior has not been described earlier (Létard *et al.*, 1998 ▸, 2003 ▸; Ksenofontov *et al.*, 1998 ▸; Capes *et al.*, 2000 ▸). It is, however, noteworthy that a visually similar two-step behavior was observed in diffuse reflectivity data at elevated hydro­static pressures and was correlated with a new phase of the compound (polymorph III) (Rotaru *et al.*, 2009 ▸).

### 3.2. Thermal properties

While DSC measurement on **Bia-P_ortho_** is reported in the literature (Létard *et al.*, 1998 ▸), providing the value of *T*_½_, Δ*H*, and Δ*S* (as listed in Table 1 ▸), DSC data for** Bia-P_mono_** have not been reported earlier. Our DSC data for **Bia-P_mono_** exhibits a broad exothermic anomaly at approximately *T*_½_ = 205.6 K upon cooling and an endothermic anomaly on heating at about *T*_½_ = 208.7 K (Fig. 4 ▸). The value of *T*_½_ is estimated as the temperature which divides the integrated area under the peak by half. The measurement was performed at a rate of 10 K min^−1^ and a thermal hysteresis is visible, similar to the observations in the magnetization data measured at fast scan rates (discussed later in Section 8).

The enthalpy associated with the spin transition corresponds to the area under the peak, and since Δ*G* = 0 at the transition temperature, the overall entropy variation upon spin transition is calculated at *T*_½_ using the relation *T*_½_ = Δ*H*/Δ*S*, by considering the limits of integration corresponding to Δ*T*_80_.^3^ The calculated entropy change Δ*S* was found to be 47 ± 2 J mol^−1^ K^−1^ and 48 ± 2 J mol^−1^ K^−1^ during the cooling and warming processes, respectively.

## 4. Crystal structure

The crystal structures of both polymorphs have been studied in detail as a function of temperature previously (Buron-Le Cointe *et al.*, 2012 ▸; Daubric *et al.*, 2000 ▸; Létard *et al.*, 2003 ▸; Marchivie *et al.*, 2003 ▸, 2005 ▸). In the following Sections 4.1 to 4.3, we therefore only describe our observations briefly, relying on the detailed descriptions given in the earlier studies.

**Figure 2:**
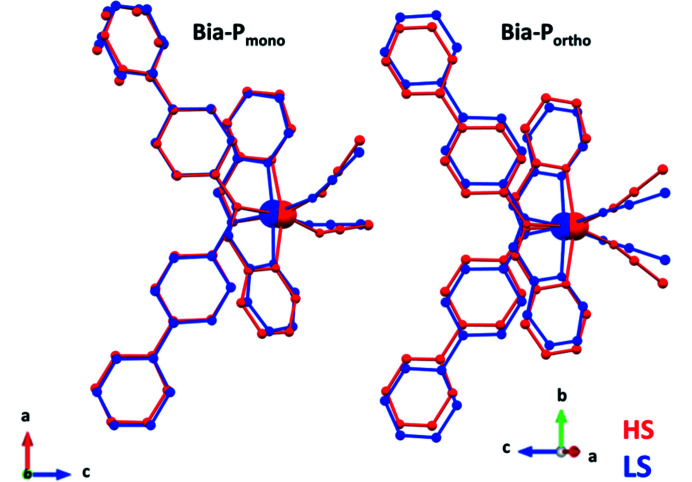
Molecular structure for the two polymorphs in the HS (red) and LS (blue) states: (left) **Bia-P_mono_** and (right) **Bia-P_ortho_**.

**Figure 3:**
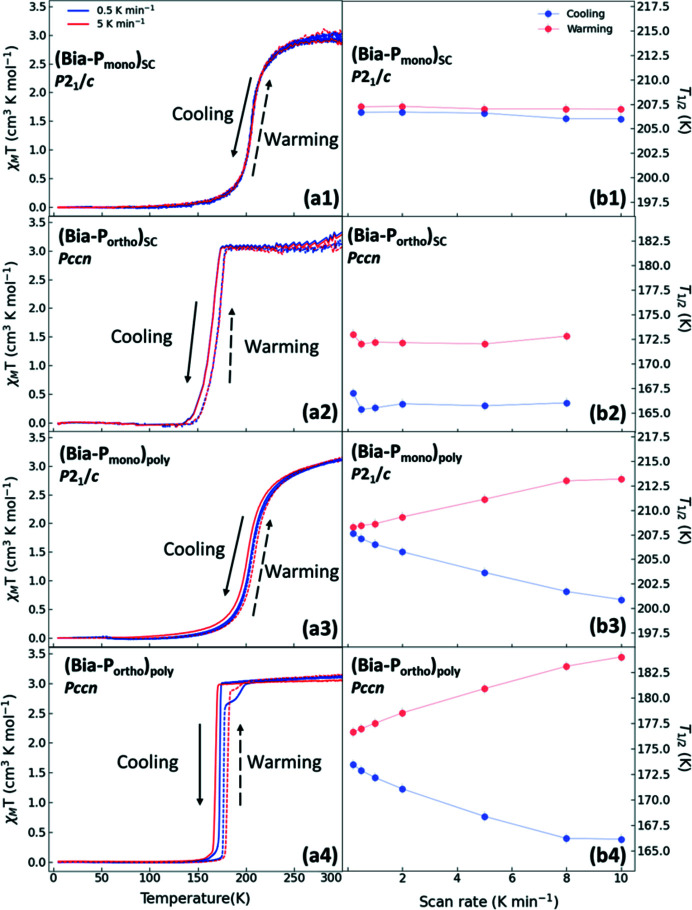
(Left) χ_M_ *T* as a function of temperature at two different scan rates at 0.5 K min^−1^ (blue) and 5 K min^−1^ (red) for **Bia-P_mono_** (views *a*1, *a*3) and **Bia-P_ortho_** (views *a*2, *a*4). (Right) The observed *T*_½_, during cooling (blue) and warming (red), as a function of the scan rate for **Bia-P_mono_** (views *b*1, *b*3) and **Bia-P_ortho_** (views b2, b4) respectively. The solid and the dashed lines correspond to the warming and cooling cycles, respectively. The abbreviations poly and SC stand for polycrystalline and single crystal, respectively.

**Figure 4:**
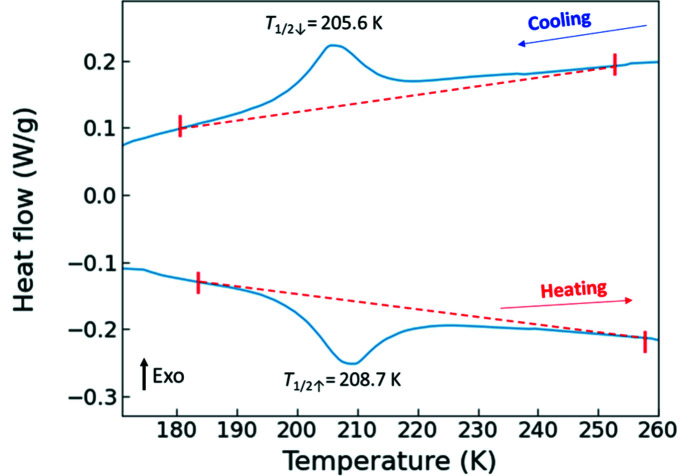
DSC data for polycrystalline **Bia-P_mono_** in cooling and heating mode at a rate of 10 K min^−1^ showing heat flow as a function of temperature processed using the instrument software. The transition temperature is given.

Figs. 1 ▸ and 2 ▸ show the crystal packing and the overlapped HS–LS crystal structures, respectively, for both polymorphs. The structures are formed by layers of molecular units (Fig. 1 ▸), arranged in the *bc* plane for **Bia-P_mono_** and in the *ac* plane for **Bia-P_ortho_**. The molecular layers are stacked for **Bia-P_mono_** along the *a* axis and **Bia-P_ortho_** along the *b* axis. The NCS^−^ groups remain almost linear, with N≡C—S angles of 179.4 (2)° for **Bia-P_ortho_** and 179.3 (3)° for **Bia-P_mono._** The main differences in the HS spin state of both compounds are:

(*a*) In **Bia-P_mono_** one of the external phenyl groups is disordered^4^ over two positions, whereas in **Bia-P_ortho_** both external phenyl groups are ordered (Fig. 2 ▸).^4^

(*b*) The Fe—N—C(S) angles for **Bia-P_mono_** diverge significantly more from linearity [154.7 (2)° and 159.0 (1)°] than in **Bia-P_ortho_** 167.7 (3)° (Fig. 2 ▸).

(*c*) Intermolecular S⋯H—C contacts are significantly shorter in **Bia-P_ortho_** [3.430 (1) Å] than in **Bia-P_mono_** [3.5126 (2) Å].

### 4.1. Temperature dependence of the unit-cell parameters and unit-cell volume

**Figure 5:**
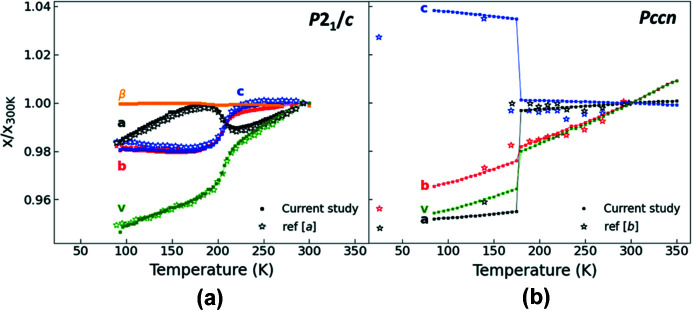
Normalized unit-cell parameters and unit-cell volume (normalized to the value at 300 K) as a function of temperature for the **Bia-P_ortho_** (right) and **Bia-P_mono_** (left). Filled symbols (black, red, and blue) are from the current study. Open star symbols are from ref [*a*] (Marchivie *et al.*, 2003 ▸), and ref [*b*] (Daubric *et al.*, 2000 ▸). Lines are guides to the eye. Error bars are the same size or smaller than the symbols.

The normalized unit-cell parameters^5^ of both polymorphs as a function of temperature are in excellent agreement with the available literature data (Fig. 5 ▸). For **Bia-P_ortho_**, the evolution of the unit-cell parameters upon cooling shows abrupt changes in a very narrow temperature region around the HS–LS transition, whereas changes in unit-cell parameters of **Bia-P_mono_** are smoother and stretch out over a larger temperature range. As expected, for both compounds, the unit-cell volume decreases at the HS–LS transitions. The decrease is significantly larger in **Bia-P_mono_** (−4.92%) than in **Bia-P_ortho_** (−3.97%) (Fig. S3). A strongly anisotropic behavior of the unit-cell parameter is observed, which is strikingly different in both compounds.

For both polymorphs (Fig. 1 ▸), within the molecular plane the thio­cyanate group points along the *c* direction. At the spin transition, *c*_mono_ decreases (−1.53%), whereas *c*_ortho_ sharply increases (+3.2%) with decreasing temperature. The other perpendicular direction within the molecular layer (*b* in **Bia-P_mono_** and *a* in **Bia-P_ortho_**) decreases in both polymorphs, although to a significantly different extent (−1.53% and −4.1% for **Bia-P_mono_** and **Bia-P_ortho_**). The unit-cell parameter corresponding to the stacking direction of the sheets (*a* in **Bia-P_mono_** and *b* in **Bia-P_ortho_**) increases at the HS–LS transition in the monoclinic phase (+0.97%), whereas it decreases in the orthorhombic phase (−0.6%).

### 4.2. Temperature dependence of intramolecular geometry

The main structural changes associated with the spin transition are at the level of the coordination sphere of the central Fe^2+^ ion (Gütlich *et al.*, 2013 ▸; Collet & Guionneau, 2018 ▸; Lakhloufi *et al.*, 2016 ▸) which is surrounded by three pairs of nitro­gen atoms: the pyridyl­methyl­ene ligand (**N_PM_**), the amino­biphenyl ligand (**N_Bia_**) and the thio­cyanate ligand (**N_CS_**) (Fig. 2 ▸).

**Figure 6:**
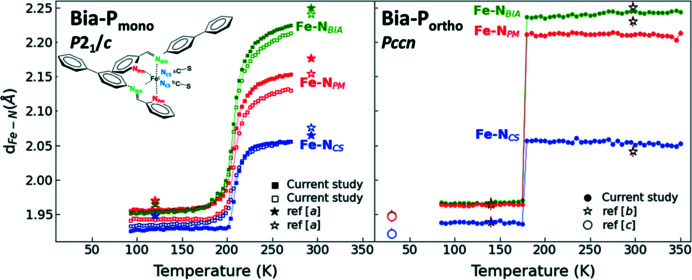
Fe—N bond length as a function of temperature for **Bia-P_ortho_** (right) and **Bia-P_mono_** (left). The square symbols (green, red, blue) are from this study. Star symbols represent the two data points from ref [*a*] (Marchivie *et al.*, 2003 ▸), ref [*b*] (Létard *et al.*, 1998 ▸), and open circles are from ref [*c*] (Guionneau *et al.*, 2001 ▸). The inset shows a schematic diagram of Fe(PM-Bia)_2_(NCS)_2_. The solid lines are guides to the eye. Error bars are the same size or smaller than the symbols.

The small temperature step that was chosen in our XRD measurements allows us to map the temperature evolution of the structural transition and clearly highlights the differences in the nature of the transition of the two polymorphs. The HS–LS transition leads to a shortening of the Fe—N distances by approximately 0.2 Å on average in both compounds (Fig. 6 ▸). The Fe—N distances are in good agreement with the two/three available data points from the literature (Marchivie *et al.*, 2003 ▸; Létard *et al.*, 1998 ▸; Guionneau *et al.*, 2001 ▸). It is particularly striking that the sharp reduction in the Fe—N distances in **Bia-P_ortho_** happens in a very narrow temperature range of about 1 K difference, between 177 K and 178 K (Fig. S4). In both polymorphs, the deviation of the Fe—N≡C(S) angles from linearity is less in the LS state than in the HS state (Fig. S5). While the degree of linearity of the thio­cyanate group (NCS)^−^ is only slightly decreased at the HS–LS transition for **Bia-P_ortho_**, more noticeable changes are observed for **Bia-P_mono_** where one of the branches is significantly less linear in the LS state and the second branch exhibits an anomalous change around the transition region (Fig. S5), which is reflected in the irregular behavior of the *a* unit-cell parameter in the transition region (Fig. S6).

In both polymorphs, the N_PM_—Fe—N_PM_ and N_Bia_—Fe—N_CS_ angles show an increase in linearity in the low-spin state (Fig. S7). N_Bia_—Fe—N_Bia_ and N_Bia_—Fe—N_CS_ angles get closer to the ideal value of 90° (Fig. S8); however, in **Bia-P_mono_**, one of the N_PM_—Fe—N_CS_ branches, which is directly related to the disordered phenyl ring, shows again anomalies around the transition temperature (Fig. S9).

Calculation of the octahedral distortion parameters, namely ζ (bond length distortion), Σ (angular distortion), and θ [the deviation from a perfectly octahedral geometry, *O*_h_, to a trigonal prismatic structure, *D*_3h_ (Ketkaew *et al.*, 2021 ▸)] reveals a higher distortion of **Bia-P_ortho_** compared to **Bia-P_mono_** in the high-spin state (see Fig. S10). During the spin state transition, the maximum relative change is observed in Δζ_HL_. In the low-spin state, the values of all the distortion parameters decrease and remain nearly constant, leading to more symmetrical octahedra.

**Figure 7:**
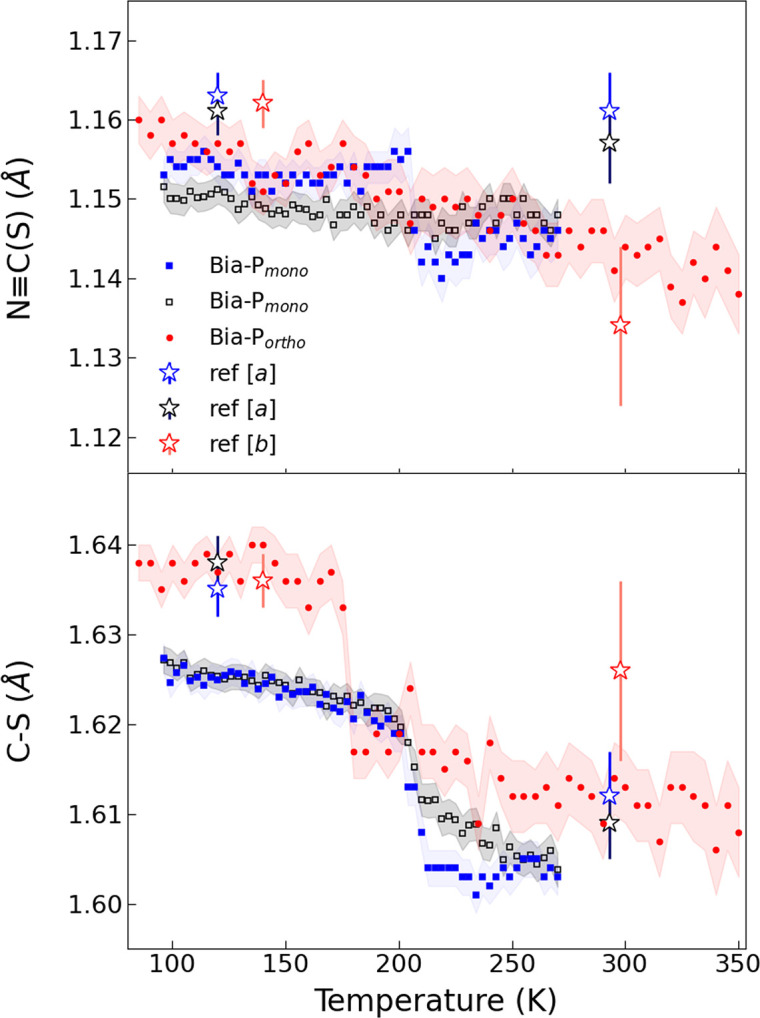
(Top) The evolution of the N≡C bond length as a function of temperature. (Bottom) The C—S bond length as a function of temperature. Red filled circles (**Bia-P_ortho_**), and filled blue and open back square symbols (**Bia-P_mono_**) are from this study. Open blue and black stars are from ref [*a*] (Marchivie *et al.*, 2003 ▸). Red open star symbols are from ref [*b*] (Létard *et al.*, 1998 ▸).

Due to the large number of temperature points and smaller standard deviations when compared with the available literature data, significant temperature-dependent changes are visible for the thio­cyanate ligand: in general, the N≡C(—S) triple bond shows an increase at the HS–LS transition, which is particularly abrupt for one of the N≡C—S branches in **Bia-P_mono_** (Fig. 7 ▸). In addition, all C—S bond lengths of the thio­cyanate ligands show an apparent increase at the HS–LS transition in both polymorphs with changes being abrupt for **Bia-P_ortho_**. Elongation of the N≡C bond length across the HS–LS transition is due to an increase in back bonding in the LS state, *i.e.* the metal donates electrons to the ligand, which results in a weakening of adjacent bonds.

As for the other ligands, most of the intramolecular distances within the pyridine and the phenyl­ene rings are only weakly influenced and stay either constant or show a slight increase with decreasing temperature (Fig. S11). However, it is worth noting that the C—C bond lengths in one of the phenyl­ene rings in **Bia-P_mono_** show significant anomalies around the transition temperature (Fig. S12).

**Figure 8:**
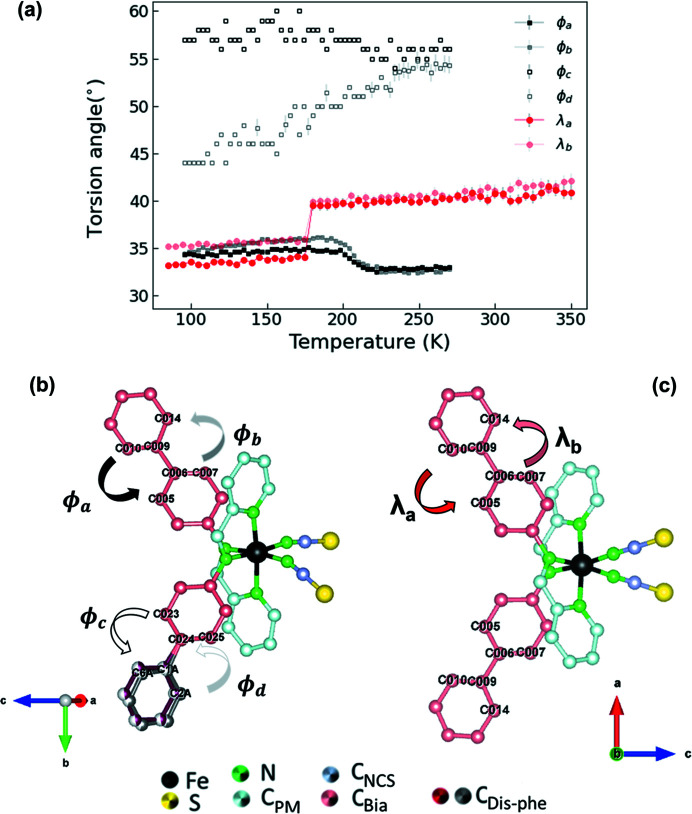
(*a*) Temperature evolution of the torsion angles of the two bi­phenyl rings for the two polymorphs (black symbols: **Bia-P_mono_**; red symbols: **Bia-P_ortho_**). Lines are guide to the eyes. Views (*b*) and (*c*) illustrate the torsion angles of **Bia-P_mono_** and **Bia-P_ortho_**, respectively.

In addition, a striking difference between both polymorphs is related to the geometry of the bi­phenyl rings. Whereas the intramolecular torsion angle of the bi­phenyl rings (defined in Fig. 8 ▸) strongly and abruptly decreases at the HS–LS transition in **Bia-P_ortho_**, following the abrupt changes in the Fe—N distances, these angles gradually increase in both bi­phenyl ligands at the HS–LS transition in **Bia-P_mono_** (Fig. 8 ▸), which is also corroborated by the anomalous behavior of the *a* unit-cell parameter (Fig. S6). In addition, within **Bia-P_mono_** the torsion angle of the bi­phenyl ring, which is affected by the disorder, is larger than the angle in the ordered bi­phenyl ligand, leading to a slight asymmetry of these two branches of the molecule.

### 4.3. Intermolecular contacts

The two polymorphs studied here are ideal for investigating the role of intermolecular interaction on the nature of the spin transition, as one of the polymorphs (**Bia-P_mono_**) shows a gradual spin transition, while the other one (**Bia-P_ortho_**) exhibits an abrupt spin transition. To describe the efficiency with which structural changes at individual spin crossover metal sites are transmitted throughout the bulk material, Slichter and Drickamer introduced a phenomenological interaction parameter, Γ, called cooperativity (Slichter & Drickamer, 1972 ▸). Earlier studies on SCO compounds suggest that the strength of the cooperativity depends directly on the strength of the π–π interaction (Guionneau *et al.*, 1999 ▸; Létard *et al.*, 1997 ▸), the van der Waals forces (Weber *et al.*, 2008 ▸; Buron-Le Cointe *et al.*, 2012 ▸; Martinez & Iverson, 2012 ▸) and the hydrogen bonding in the system (Real *et al.*, 2003 ▸; Shen *et al.*, 2019 ▸).

In general, π–π interactions can be classified into three categories based on the interplanar angle and on the distances between the centroids of aromatic rings: strong, moderate and weak interactions (Martinez & Iverson, 2012 ▸). An investigation on the strength of these interactions with the *Mercury* (Macrae *et al.*, 2020 ▸) program shows that for **Bia-P_ortho_**, no strong π–π interaction between the phenyl rings is observed, neither in the HS nor in the LS state.

For **Bia-P_mono_**, on the other hand, a number of strong π–π interactions between two phenyl rings are observed, and they are similar in the LS and in the HS state. In addition, in the transition region at ∼225 K, some of the moderate π–π interactions become stronger, while at lower temperatures, these interactions are weakened again (Fig. S13, Table S2). As these interactions point along the *a* direction of the crystal structure, they contribute to the observed anomalies in the temperature dependence of the *a* unit-cell parameter.

Both polymorphs show short C⋯C contacts [< 3.4 Å, *i.e.* smaller than the sum of van der Waals radii (Batsanov, 2001 ▸)], suggesting the presence of van der Waals interactions in the system. Of these, the shortest C⋯C contacts [3.326 (11) Å] are observed in **Bia-P_mono_** in the HS state and they also persist in the LS states. The short C⋯C contacts in the case of **Bia-P_mono_** lead to the formation of a three-dimensional network of strong van der Waals interactions in this polymorph. For **Bia-P_ortho_**, fewer C⋯C contacts are observed, which are weaker in the HS state than the ones observed in **Bia-P_mono_** [3.428 (9) Å], yet some of them become stronger in the LS state [3.324 (9) Å].

**Figure 9:**
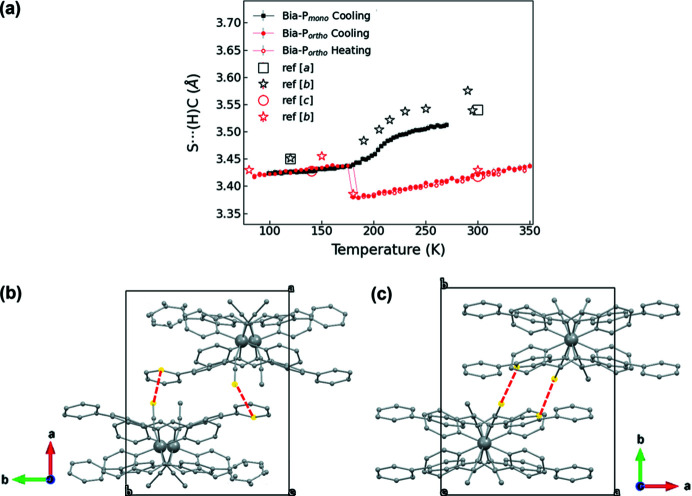
(*a*) Evolution of the shortest intermolecular S⋯C distance for **Bia-P_ortho_** (small filled and open red circles while cooling and heating, respectively) and **Bia-P_mono_** (black squares). Views (*b*) and (*c*) show the S⋯C shortest distance between two molecules in **Bia-P_mono_** and **Bia-P_ortho_**, respectively. Open large symbols are from ref [*a*] (Marchivie *et al.*, 2003 ▸), ref [*b*] (Buron-Le Cointe *et al.*, 2012 ▸) and ref [*c*] (Guionneau *et al.*, 2001 ▸). The solid lines in (*a*) are guides to the eye. Error bars are the same size or smaller than the symbols.

The third important interaction which might influence the cooperativity is the intermolecular hydrogen-bonding interaction involving the sulfur atoms of the NCS^−^ branches with the closest (H)—C atom in one of the internal bi­phenyl rings. For **Bia-P_ortho_**, a short S⋯ (H)—C contact [less than 3.5 Å (Batsanov, 2001 ▸)] exists in the HS state, whereas for **Bia-P_mono_**, the shortest contact in the HS state is larger than 3.5 Å, and a value less than 3.5 Å is only reached at lower temperatures (Fig. 9 ▸). It is striking that the length of the intermolecular S⋯C contacts for both polymorphs becomes almost identical in the LS state due to an increase of this distance when **Bia-P_ortho_** passes the HS–LS transition.^6^ As the S⋯(H)—C contacts show no significant change in bond lengths, we assume that the hydrogen-bonding network is not substantially changed during cyclic measurements.

Intermolecular interactions can be classified into two categories: those within the molecular plane, the so-called intrasheet contact, and those that involve molecular units belonging to different sheets called intersheet contact. For **Bia-P_mono_** in the HS state, several intra- and intersheet contacts correspond to van der Waals interactions. For **Bia-P_ortho_**, in the HS state, there is no intrasheet contact corresponding to van der Waals interactions, yet there is one intersheet contact which corresponds to hydrogen bonding. In the LS state of both polymorphs, the intersheet contacts are formed by hydrogen bonding, whereas the intrasheet contacts correspond to van der Waals interactions. The variation of the various intra- and intersheet contacts upon cooling is different with some of them decreasing through the HS–LS transition and others increasing (Fig. S15). It is worth noting that due to the lower symmetry of **Bia-P_mono_**, the nature of the intermolecular contacts is different on the two sides of the molecular layers.

## 5. Coexistence of HS and LS domains, HS and LS states, and atomic displacement parameters

**Figure 10:**
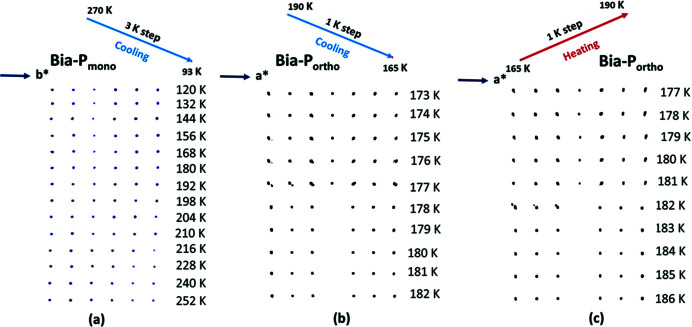
Temperature dependence across the HS to LS transition of a line of diffraction spots: (*a*) **Bia-P_mono_** upon cooling along (0, *k*, 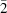 ) with *k* varying from 7 to 12; **Bia-P_ortho_** upon cooling (*b*) and heating (*c*) along (*h*, 0, 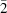 ) with *h* varying from −3 to 3. Note the different temperature scales.

Reconstructions of reciprocal space based on single-crystal diffraction data (Fig. 10 ▸) for **Bia-P_ortho_** show a splitting of the Bragg peaks in the region of the spin crossover transitions, which can be attributed to the formation and coexistence of domains of the HS and LS states, hallmarks of a first-order transition. The superposition of the crystal structures corresponding to the two spin states results in the observed splitting of the Bragg reflections. The intensities corresponding to the two states were integrated together. As a consequence, in the refinement an increase in the ADP values of the atoms is visible, which is clearly seen, in particular for the Fe atom (see Fig. S4).

On the other hand, for **Bia-P_mono_**, the gradual crossover from HS to LS leads to a continuous shift of positions of the Bragg peaks. This indicates that in this polymorph the HS and LS states are not distributed into larger domains, but instead there is probably a random distribution of HS/LS molecules throughout the crystal.

**Figure 11:**
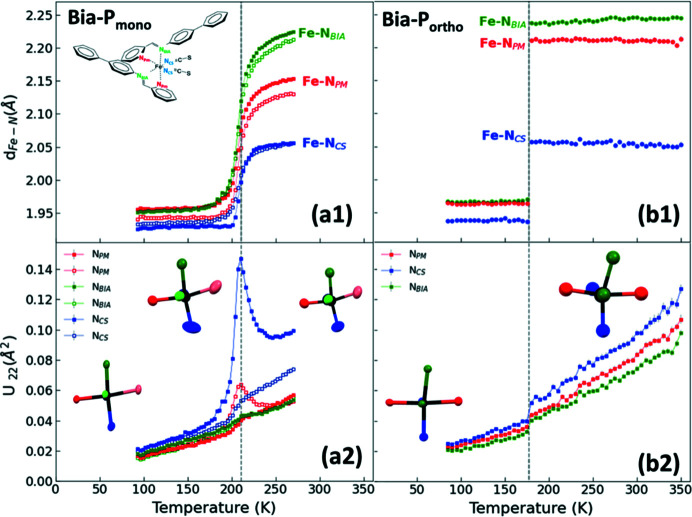
Temperature dependence of *U*_22_ (Å^2^) for the N atoms of (*a*) **Bia-P_mono_** and (*b*) **Bia-P_ortho_**. The gray dashed line represents the transition temperature. The schematic diagram insets in (*a*1), (*a*2) and (*b*2) show the molecular structure of the Fe(PM-Bia)_2_(NCS)_2_ and the corresponding ADPs of the N atoms bonded to Fe atom, respectively.

This is reflected in the temperature dependence of the ADPs of the N atoms of **Bia-P_mono_** (Fig. 11 ▸). Outside the SCO range, the ADPs exhibit a monotonic decrease with decreasing temperature. Around the transition, a clear λ-type anomaly is observed for *U*_22_ of two of the nitro­gen atoms, in particular for the one attached to the thio­cyanate group at *T*_½_, which indicates a large displacement in a direction perpendicular to the Fe—N bond (see Fig. 11 ▸ and inset therein). It should be noted that some SCO compounds exhibit anomalous ADPs of the N atoms along the Fe—N bonds (Chernyshov *et al.*, 2009 ▸). This anomaly is a consequence of the fact that disorder is present at *T*_½_ with half of the Fe^2+^ cations in the high-spin state and the other half in the low-spin state. As the instrumental resolution of the diffraction experiment is only 0.8 Å, the disorder cannot be resolved but is instead modeled in terms of the average between HS and LS positions, and the disorder contribution to the atomic displacement parameter (Chernyshov *et al.*, 2003 ▸). For **Bia-P_mono_** the ADPs normal to the Fe—N bond are not sensitive to the disorder in Fe—N distances. The observed increase of the *U*_22_ parameter of nitro­gen near *T*_½_ might be due to a disordered component related to the difference in angles and tilting of the entire complex for two co-existing spin states.

## 6. Thermal cycling

A systematic analysis of the reproducibility of the spin transition as a function of temperature is of fundamental importance for the future caloric application of a material. To elucidate this aspect, we carried out consecutive cooling and heating cycles, both for the magnetic susceptibility and X-ray diffraction measurements.

**Figure 12:**
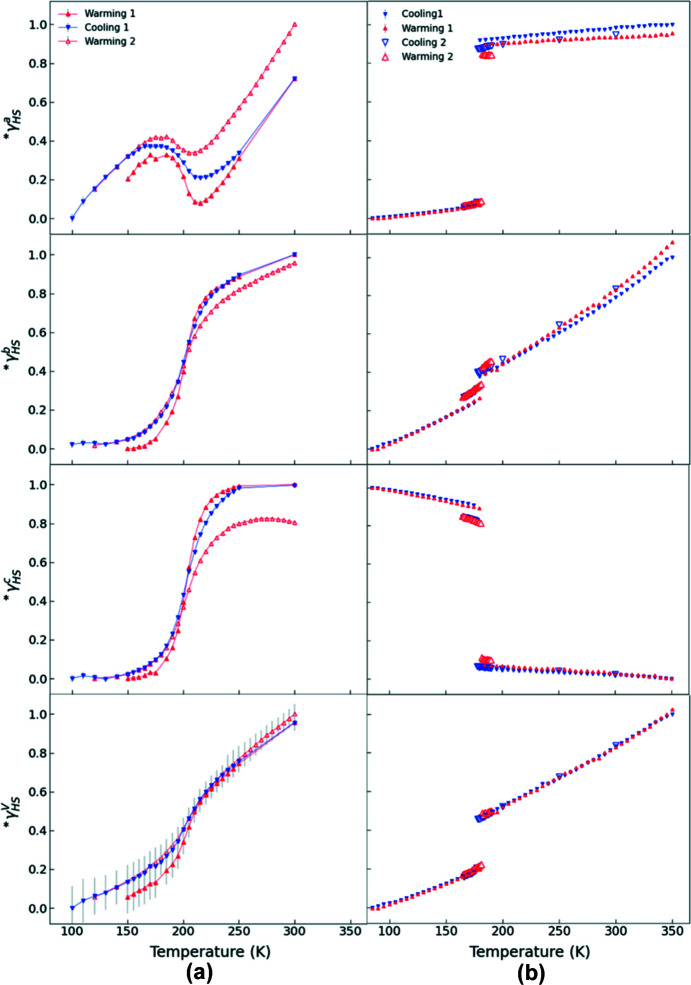
Evolution of relative change in the high-spin fraction calculated on the basis of unit-cell parameters and unit-cell volume upon consecutive thermal cycles for **Bia-P_ortho_** and** Bia-P_mono_** (right and left, respectively). Calculated using 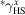 = [*x*(LS)_cycle1_ − *x*(*T*)][*x*(LS)_cycle1_ − *x*(HS)_cycle1_]^−1^. Filled blue and red triangle symbols represent the value of the first cooling, followed by the first warming, respectively. Open blue and red triangles represent the second cooling followed by the second warming. Lines are guides to the eyes. Error bars are the same size or smaller than the lines.

The magnetic susceptibility measurement, which was carried out for **Bia-P_mono_** in two consecutive cycles (warming, cooling, warming, cooling and warming again), indicates a nearly perfect reproducibility. On the other hand, the unit-cell volume and unit-cell parameters (Fig. 12 ▸) extracted from the diffraction data are not perfectly reproducible on cycling. The volume in the second warming cycle increases by about 0.3% compared to the first warming cycle, whereas the unit-cell parameters show different deviation in the LS and HS states with respect to the first warming cycle.

Despite a significant change in volume, the unit-cell volume of **Bia-P_ortho_** is perfectly reproduced in all measured cycles using X-ray diffraction (2× cooling and 2× warming; Fig. 12 ▸), although the same is not true for the behavior of individual unit-cell parameters. In the first cooling and warming cycle, all the unit-cell parameters are fully reproducible in the LS state, yet they are not in the HS state. During the second warming and cooling cycle, all the unit-cell parameters are badly reproduced both in the HS and LS states. As the parameters describing the mosaicity of the crystal exhibit a nearly constant and perfectly reproducible behavior on cycling (Fig. S16), indicating that the crystal maintains its quality, the observed changes must be attributed to underlying changes in the crystal structure.

On careful inspection of the structural and the intra–intermolecular features (obtained from the single crystal data), during the cyclic measurements for **Bia-P_ortho_**, they do not exhibit a significant change when taking into account the resulting standard deviations (see Fig. S5). Thus, the overall change of the unit-cell parameters must be a consequence of several very small changes in the crystal structure across the lattice, which add up to the observed differences on cycling.^7^

## 7. Entropy changes and cooperativity

The spin crossover behavior is characterized by the high-spin fraction (γ_HS_), which is also the order parameter for the HS↔ LS transition (Gütlich *et al.*, 2013 ▸; Chernyshov *et al.*, 2004 ▸). It can be obtained from the crystal structure data, via the Fe—N bond lengths, and from the magnetization data, via the magnetic susceptibility χ_M_, using the following expressions,

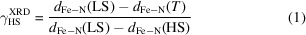




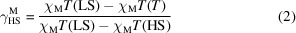




An estimate of the thermodynamic parameters, the enthalpy (Δ*H*) and entropy (Δ*S*) associated with the spin-crossover, could be obtained by fitting the values of γ_HS_, obtained from equations (1) and (2), to the Slichter–Drickamer (1972 ▸) model:

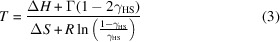




Here the parameter Γ corresponds to the cooperativity, which is assumed to be temperature independent and traces its origin to the interaction between individual spins and the average magnetization of the crystal (Halcrow, 2013 ▸; Kreutzburg *et al.*, 2017 ▸). Further *R*, Δ*H* and Δ*S* denote the universal gas constant, the enthalpy and entropy changes associated with the HS↔LS transition, respectively. At the equilibrium temperature *T*_½_, corresponding to γ_HS_ = 0.5 (Nicolazzi & Bousseksou, 2018 ▸), the enthalpy in equation (3) can be substituted with the relation Δ*H* = Δ*S* *T*_½_, allowing the modification of equation (3) as:

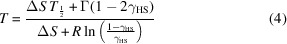




**Figure 13:**
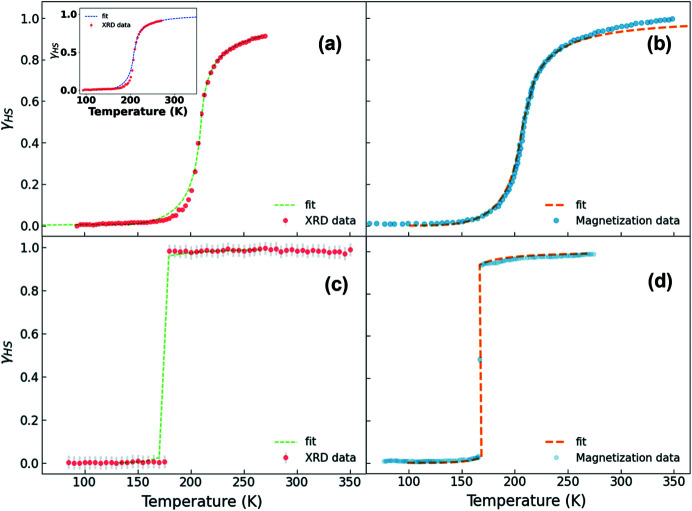
HS fraction γ_HS_ as a function of temperature for **Bia-P_mono_** (*a*,*b*), and **Bia-P_ortho_** (*c*,*d*). The red circles are obtained from crystal structure data. Blue circles indicate the magnetization data. The dashed line corresponds to fitting of the Slichter–Drickamer model [see equation (4)]. The inset in (*a*) shows the fit of the data obtained from crystal structure data fitted with the Slichter–Drickamer model, yet fixing the thermodynamic values to the ones determined by DSC measurement.

Using equation (4), a fit was carried out for γ_HS_, keeping Δ*S* and Γ as the variable parameters. For **Bia-P_mono_** and **Bia-P_ortho_**, Figs. 13 ▸(*a*), 13 ▸(*c*) illustrate the fit obtained from crystal structure data and Figs. 13 ▸(*b*), 13 ▸(*d*) show the corresponding fit from magnetization data, respectively.

The value of cooperativity (Γ) obtained from the single-crystal structural data could be further confirmed using the DSC measurements. For this, using the value of Δ*S* obtained from the DSC data, a fit of γ_HS_ obtained from the Fe—N bond lengths was carried out. The value of Γ obtained from the fit was found to be in good agreement with the one obtained on considering both Δ*S* and Γ as free parameters.

Due to the continuous nature of equation (4), a fit to the abrupt and discontinuous magnetization and bond length curves for **Bia-P_ortho_**, cannot be obtained. We thus fixed the value of Δ*S* deduced from DSC measurements (Létard *et al.*, 1998 ▸) and allowed only Γ as the variable parameter [Fig. 13 ▸(*c*)].

The values obtained from the above analysis are summarized in Table 1 ▸. The value of Δ*S* is significantly larger than the entropy variation resulting from the change of the spin state Δ*S*_ele_ = *R*ln[(2*S*_HS_ +1)/(2*S*_LS_ +1)] = 13.4 J mol^−1^ K^−1^ (for *S*_HS_ = 2 and *S*_LS_ = 0). The excess entropy can be attributed to the contribution from vibrational, configurational and rotational degrees of freedom present in both polymorphs (Molnár *et al.*, 2019 ▸). The obtained values of Γ and *T*_½_ for both polymorphs are in good agreement with the Slichter–Drickamer model, which attributes the gradual transitions to weak interactions with Γ < 2*R* *T*_½_ and abrupt transitions to strong interactions with Γ > 2*R* *T*_½_ (see Table 1 ▸) (Nicolazzi & Bousseksou, 2018 ▸).

## 8. Discussion

The detailed crystallographic study of the temperature dependence of the two polymorphs shows that while changes in structural parameters of **Bia-P_ortho_** happen in a ‘one-step’ mechanism in a small temperature interval, for **Bia-P_mono_** the observed behavior is not only stretched out over a large temperature interval of the gradual spin transition, but it is also more complex. Several structural parameters (*e.g.* the *a* unit-cell parameter) show changes of trend in the region of the transition, which correspond to changes, both in the intramolecular geometry and in the intermolecular interactions, in **Bia-P_mono_**. In addition, the comparison of reciprocal space sections and atomic displacement parameters of both polymorphs suggest that while in **Bia-P_ortho_** the microstructure in the transition region is characterized by the existence of HS and LS state domains, in **Bia-P_mono_** there is most probably rather a random distribution of molecular complexes, which are in the HS or LS states.

With the help of the Slichter–Drickamer (1972 ▸) model, the enthalpy (Δ*H*) and entropy (Δ*S*) associated with the spin crossover were extracted from the magnetization, DSC and crystal structure data. The cooperativity Γ was also extracted using this model, with higher Γ for **Bia-P_ortho_** in agreement with the abruptness of the SCO transition of this polymorph.

Unfortunately the Slichter and Drickamer model, and in general any thermodynamic model, does not clarify the atomistic origin of cooperativity. To elucidate the relationship between the thermodynamic parameters, the distinct nature of the transitions and the crystal structures, it is useful to discuss the intermolecular interactions. In the literature, it is frequently assumed that the extent of cooperativity increases with the number and strength of intermolecular interactions (Marchivie *et al.*, 2003 ▸; Hayami *et al.*, 2003 ▸; Guionneau, 2014 ▸). However, there are also examples where a large number of short intermolecular contacts inhibit the propagation of the structural changes associated with the spin crossover (Halcrow, 2011 ▸; Reger *et al.*, 2005 ▸).

A detailed comparison of the intermolecular interactions in the two polymorphs investigated here shows that a differentiated view on the interactions is necessary, as, on the one hand, **Bia-P_mono_** has stronger van der Waals and π–π interactions than **Bia-P_ortho_**, and, on the other hand, hydrogen bonding is only observed in the HS state of **Bia-P_ortho_** and is absent in the HS state of **Bia-P_mono._** The cooperativity is significantly higher for the HS–LS transition in **Bia-P_ortho_** and thus seems to be mainly due to the hydrogen bonding between the molecular units which serve as a means to transmit the structural deformations associated with SCO throughout the lattice in a highly cooperative manner. The absence of π–π and van der Waals interactions in **Bia-P_ortho_** might then even provide more freedom at the intramolecular level to propagate these changes throughout the lattice. In **Bia-P_mono_**,the stronger van der Waals interaction and π–π interaction are possibly competing and lead to smearing out of the transition over a large temperature range. An examination of number and strength of intermolecular contacts may therefore be insufficient to predict the cooperativity of spin conversion, as different contacts contribute to the cooperativity with different signs.

**Figure 14:**
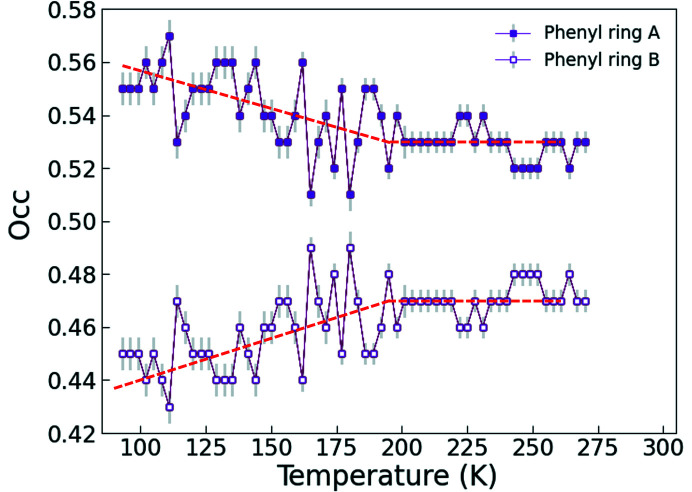
Temperature dependence of the occupation fraction of the two disorder positions of the phenyl group in **Bia-P_mono_**.

The disordered phenyl group in **Bia-P_mono_** has nearly equal probabilities of occupation of the two positions *A* and *B* in the high-spin state. Below the spin transition temperature, one of the two positions shows an increased probability of occupation over the other (Fig. 14 ▸). This indicates that the configurational disorder might play a role in the entropy changes during the spin-crossover transition.

The observed influence of thermal cycling on both polymorphs can also be directly related to the cooperativity. While magnetization measurements are found to be reproducible for both polymorphs, the same is not true when the structural parameters, measured with synchrotron light, are considered. For **Bia-P_ortho_** the unit-cell volume is reproducible upon cycling and the crystal does not exhibit any apparent deterioration (cracks, fractures), demonstrating its exceptional robustness upon cycling, which can be related to the strong intermolecular contacts. On the other hand, for **Bia-P_mono_** the unit-cell volume increases on cycling (Fig. 12 ▸). This can be related to radiation damage in which the dose is accumulated progressively with time and temperature (Chernyshov *et al.*, 2022 ▸). The radiation dose seems to affect the spin-crossover process and favors the HS state, shifting the transition region to lower temperatures (from 210.6 K to 208.8 K).

The apparent rate-dependent hysteresis in the magnetization data, observed for the polycrystalline samples of both polymorphs, could be related to the temperature lag between the sample and the temperature controller which increases with higher scan rates, leading to broadening of the thermal hysteresis. The fact that a similar effect is not observed for the single crystals rather points to a grain size dependent phenomenon. A possible explanation might be linked to the grain size dependent intrinsic kinetic behavior of the domain formation (Ridier *et al.*, 2018 ▸).

For the slowest scan rates, in the polycrystalline sample the thermal hysteresis still exists for **Bia-P_ortho_** (hysteresis ≃ 4 K), while for **Bia-P_mono_** the thermal hysteresis almost vanishes. This can be explained on the basis of the intrinsic kinetics of the spin conversion, which is slower than the temperature change for **Bia-P_ortho_**. However, due to the gradual nature of transition, a slow scan rate allows more time for **Bia-P_mono_** to overcome the barrier between the LS and HS states (see appendix A1[App appa]).

An important difference in the kinetic behavior of the two polymorphs is the formation of a scan rate dependent intermediate state on warming for **Bia-P_ortho_**, which is absent for **Bia-P_mono_**. The similarity between our observations for low scan rates and the state observed at hydro­static pressures above 1 kbar (which was attributed by the authors to the co-existence of **Bia-P_ortho_** with unknown polymorph III) (Rotaru *et al.*, 2009 ▸) is striking and deserves further investigation. For this, further diffraction experiments are required; however, these are out of the scope of this article.

## 9. Conclusion

A comprehensive study of the mechanism of the transition in the orthorhombic and monoclinic polymorphs of the spin crossover compound [Fe(PM-Bia)_2_(NCS)_2_], using magnetization, DSC and synchrotron single-crystal X-ray diffraction, is presented and the explicit role of the hydrogen bonding, π–π and van der Waals interactions on the cooperativity of the transition is highlighted. Based on the atomistic insights obtained from single-crystal diffraction, the role of inter- and intramolecular interactions and their interplay with the anisotropy of the unit-cell parameters, various interactions and symmetry of the crystal across the spin crossover has been explored. The analysis of the data highlights the complexity of the structural processes involved in the spin crossover transition. Our data are analyzed within the framework of the Slichter and Drickamer model to obtain the values of enthalpy, entropy and cooperativity associated with the spin crossover for both polymorphs, which may be utilized as experimental constraints for theoretical calculations.

In the cyclic measurements, **Bia-P_ortho_** exhibits robust reproducibility, while for **Bia-P_mono_** the effect of radiation damage is observed for longer exposure times, which induces a shift in the spin crossover temperature. While knowledge of reproducibility is vital to understand the intrinsic effects of SCO, the scan rate dependence plays an important role in applications that involve a time-dependent use of SCO compounds. A close examination of the scan rate dependence of the thermal hysteresis highlights the difference in the intrinsic nature of spin state dynamics and reveals a grain size dependence of the magnetic properties, thermal exchange and kinetic behavior for both polymorphs. Based on this observation, a theoretical model is presented to describe the key role of non-equilibrium spin state dynamics and the dependence of thermal hysteresis on the scan rate. A new scan rate dependent intermediate state appears in the heating cycle for the orthorhombic polymorph, which has not been reported previously. Further diffraction experiments are mandatory to understand the microscopic picture of this state.

The results of this paper serve as a step in understanding the nature of transition in spin crossover complexes highlighting the role of various interactions and their correlation with the microscopic features of the crystal structures. Since the nature of the transition is strongly interlinked with the atomistic features, one can potentially engineer SCO not only by tuning the number and strengths of various interactions, but also taking into account opposite signs of their contributions in the collective phenomena.

## Supplementary Material

Crystal structure: contains datablock(s) Bia_dw4400_085K, Bia_dw4400_350K. DOI: 10.1107/S2052520623005814/ne5010sup1.cif

Structure factors: contains datablock(s) Bia_dw4400_085K. DOI: 10.1107/S2052520623005814/ne5010Bia_dw4400_085Ksup2.hkl

Structure factors: contains datablock(s) Bia_dw4400_350K. DOI: 10.1107/S2052520623005814/ne5010Bia_dw4400_350Ksup3.hkl

CIF files of the monoclinic polymorph (Bia-Pmono) named as Bia_Pmono_00TK.cif representing a series of 55 temperature data sets. The measurements were carried out during cooling from 270 K to 93 K with a 3 K step. Each file follows the naming convention Bia_Pmono_00TK.cif, where T signifies the temperature in K. DOI: 10.1107/S2052520623005814/ne5010sup4.zip

CIF files of the orthorhombic polymorph (Bia-Portho) named as Bia_Portho _00TK.cif representing a series of 53 temperature data sets. The measurements were performed while cooling from 350 K to 85 K with a 5 K step. Each file follows the naming convention Bia_Portho _00TK.cif, where T represents the temperature in K. DOI: 10.1107/S2052520623005814/ne5010sup5.zip

Figures S1-S17, and Tables S1 and S2. DOI: 10.1107/S2052520623005814/ne5010sup6.pdf

CCDC references: 2278926, 2278927

## Figures and Tables

**Figure 15:**
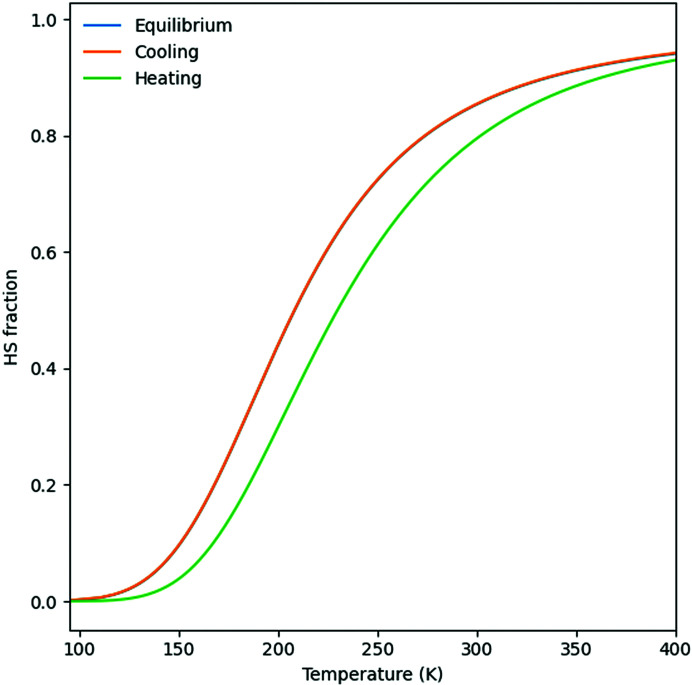
An apparent hysteresis – difference between cooling and heating regimes – for a low-cooperativity spin crossover, with parameters taken from Table 1 ▸ (crystal structure data), *A*/β ∼0.025 and *E*_a_∼1000.

**Table 1:** *T*_1/2_ and thermodynamic parameters for **Bia-P_mono_** and **Bia-P_ortho_** obtained from the crystal structure, magnetization, and DSC calorimetry measurements by fitting the data to the Slichter–Drickamer model The Δ*S*, Δ*H*, *T*_1/2_ and Γ values estimated from the magnetic and crystal structure data for **Bia-P_mono_** are within the confidence intervals of 45.0–49.4 J K^−1^ mol^−1^, 9.5–10.3 kJ mol^−1^, 206.8–210.9 K and 2.2–3.4 kJ mol^−1^, respectively.

	Thermodynamics values	Δ*S* (J mol^−1^ K^−1^)	Δ*H* (kJ mol^−1^)	*T*_1/2_ (K)	Γ (kJ mol^−1^)	2*RT*_1/2_ (kJ mol^−1^)
Monoclinic (**Bia-P_mono_**)	DSC	48 (3)	10.2 (9)	208.5 (5)	2.85 (2)	3.46 (9)
	Magnetization	47.3 (1)	9.9 (1)	208.3 (9)	2.6 (3)	
	Crystal structure	46.3 (5)	9.7 (7)	209.8 (2)	3.1 (2)	
Orthorhombic (**Bia-P_ortho_**)	Crystal structure	59†	10†	177.0 (5)	5.0 (1)	2.94 (5)

† Reference: Létard *et al.* (1998 ▸).

## Footnotes

1 The large field applied in the case of **Bia-P_ortho_** was necessary to obtain a reliable signal for the sample. Additional measurements on both samples in different fields showed no significant field effects, apart from small shifts in *T*_½_ (< 1 K *T* ^−1^).

2 After the second run, the crystal was taken out at 330 K and stored for 48 h at room temperature.

3 Δ*T*_80_ is defined as the temperature range covering 80% of the spin transition, starting from the point where 90% of the molecules are in the HS state at high temperature, to the point where 10% are in the HS state at low temperature.

4 This disorder was taken into account during the structural refinement using two positions for the phenyl ring and related fragment population parameter. Restraints on the anisotropic displacement of the disordered phenyl ring were applied.

5 We observe an offset in the absolute values of the unit-cell parameter, which is, however, not unusual for data originating from different instruments.

6 The sharp increase of the S⋯C contact in **Bia-P_ortho_** is probably closely related to the fact that the N_CS_—Fe—N_CS_ angles of the two thio­cyanates are strongly decreased at the HS–LS transition (Fig. S14), which in turn might lead to the significant increase of the contacts between thio­cyanate and carbon atoms of the neighboring phenyl rings.

7 For **Bia-P_mono_**, diffraction measurements on cycling were carried out on polycrystalline samples, where the evolution of the intermolecular changes is even more difficult to trace due to the larger standard deviations on the parameters.

## Appendix A Non-equilibrium kinetics for SCO

### A1. Non-isothermal kinetics of spin conversion

If the rate of temperature change is slow, then the system reaches equilibrium faster than temperature varies. Therefore, an equilibrium fraction of HS states can be measured, γ_eq_ (*T*). If we change the temperature faster than the system can reach equilibrium, we measure a non-equilibrium fraction of HS states, γ_ne_ (*T*).

First, we assume that we start from room temperature (*T*_0_ = 300 K) and pure HS state [γ_eq_ (*T*_0_) = 1] and then carry out cooling with ramp rate β and instant measurements of the HS fraction. Second, we introduce a normalized measure of spin conversion:

The rate of spin conversion is:

and

where *E*_a_ is the activation energy, *A*(*T*) is a complex function summing up phonon contributions for modes interacting with the spin state (Klinduhov *et al.*, 2010 ▸). (1 − α) ensures that kinetics stop when equilibrium is reached. Separating variables and integrating gives the following expression for a non-isothermal measure of spin conversion:

Denoting integral on the right side as *I* (*T*, *T*_0_, *E*_a_) and taking into account that:

A non-equilibrium fraction of HS state as a function of temperature measured on cooling reads:

or

For a heating branch we start from pure LS state:

Correspondingly, a non-equilibrium fraction of HS state as a function of temperature measured on heating reads:

Obviously, for very low ramp rates, both cooling and heating branches merge with the equilibrium curve. Note that the above equations are derived assuming that *A* does not depend on temperature and spin fraction; according to Boukheddaden *et al.* (2000 ▸), it assumes that cooperativity is neglected.

An illustration of equations (A1.9) and (A1.10) is given in Fig. 15 ▸, where the cooling curve is superimposed on the equilibrium spin fraction; this indicates a difference between kinetics in cooling and heating regimes that have to start in different temperature conditions. The simple model presented above cannot therefore completely explain a symmetric expansion of the apparent hysteresis observed experimentally (Fig. 3 ▸). Thus, one has to assume a temperature dependence for *A*, which is an *a priori* unknown function, summing up contributions for phonon modes interacting with spin states (Boukheddaden *et al.*, 2000 ▸; Klinduhov *et al.*, 2010 ▸). Its explicit form can be found experimentally by measuring kinetics of the relaxation of thermally quenched HS states within the temperature range of spin crossover.
