# Supplementary material for: Structural insight into the cooperativity of spin crossover compounds
Source: Acta Crystallogr B Struct Sci Cryst Eng Mater. 2023 Aug 11;79(Pt 5):354–67. doi: 10.1107/S2052520623005814 (PMC10552598; doi:10.1107/S2052520623005814)

STRUCTURAL SCIENCE
CRYSTAL ENGINEERING
MATERIALS

Volume 79 (2023)

Supporting information for article:

Structural insight into the cooperativity of spin crossover compounds

H. Shahed, N. Sharma, M. Angst, J. Voigt, J. Perßon, P. Prakash, K.W. Törnroos, D. Chernyshov, H. Gildenast, M. Ohl, G. Saffarini, A Grzechnik and K. Friese

Supporting information

Table S1 : Selected experimental crystal data for both polymorphs Fe(PM-BiA)$_2$(NCS)$_2$ in the two spin states (HS & LS).

	**Bia-P$_{mono}$**		**Bia-P$_{ortho}$**	
Chemical Formula	$FeN_6S_2C_{38}H_{28}$			
Temperature (K)	270	93	350	85
a (Å)	17.3421(2)	17.1496(2)	12.9662(8)	12.3332(2)
b (Å)	12.48360(10)	12.26250(10)	15.3404	14.6757(2)
c (Å)	17.2399(2)	16.9243(2)	17.5869(8)	18.2772(2)
β (°)	115.7397(15)	115.8137(16)	90	90
V (Å^3)	3361.96(7)	3203.98(7)	3498.2(3)	3308.14(8)
Density (g cm^{-1})	1.3606	1.428	1.308	1.383
μ (mm^{-1})	0.456	0.4695	0.439	0.454
Radiation type	Synchrotron			
Diffractometer	Multipurpose PILATUS@SNBL diffractometer			
No. of measured	25238	23716	36859	32190
symmetry independent	7835	7435	6983	6472
observed [I>2σ(I)] reflections	6678	6986	2327	4872
R$_{int}$	0.0313	0.0351	0.1024	0.0703
(sinθ/λ) (Å^{-1})	0.67	0.67	0.84	0.83
No. of parameters	457	457	213	213
R[F^2>2σ(F^2)]	0.0393	0.0334	0.1137	0.0643
wR(F^2)	0.1137	0.0937	0.1938	0.118

Table S2 π-π interaction of the two polymorphs as identified using the Mercury software (MacRae *et al.*, 2020). Centroid to centroid distances (D) and their relative angle of orientation (∢) are shown, both of which are used to estimate the strength of the interactions. IP stands for intermediate points; IP1 = 180 K (**Bia-P$_{ortho}$**), = 219 K (**Bia-P$_{mono}$**), IP2 = 170 K (**Bia-P$_{ortho}$**), = 180 K (**Bia-P$_{mono}$**). 'No.' stands for the number of π-π interactions.

	**Strong**												**Intermediate**											
	**HS**			**IP1** 180 K (*Pccn*) 219 K ($P2_1/c$)			**IP2** 170 K (*Pccn*) 180 K ($P2_1/c$)			**LS**			**HS**			**IP1** 180 K (*Pccn*) 219 K ($P2_1/c$)			**IP2** 170 K (*Pccn*) 180 K ($P2_1/c$)			**LS**		
	**No.**	**D (Å)**	**∡ (°)**	**No.**	**D (Å)**	**∡ (°)**	**No.**	**D (Å)**	**∡ (°)**	**No.**	**D (Å)**	**∡ (°)**	**No.**	**D (Å)**	**∡ (°)**	**No.**	**D (Å)**	**∡ (°)**	**No.**	**D**	**∡ (°)**	**No.**	**D (Å)**	**∡ (°)**
Pccn	0			0			0			0			2	5.18	35.47	2	5.16	34.24	3	5.51	18.26	3	5.45	17.22
														5.1	58.27		4.95	56.71		4.96	33.27		4.95	32.86
																				4.35	42.58		4.29	41.5

<table>
<tr><td rowspan="5">$P2_1/c$</td><td rowspan="5">1</td><td rowspan="5">4.11</td><td rowspan="5">0</td><td rowspan="5">2</td><td rowspan="2">4.09</td><td rowspan="2">0</td><td rowspan="5">1</td><td rowspan="5">4.2</td><td rowspan="5">0</td><td rowspan="5">1</td><td rowspan="5">4.13</td><td rowspan="5">0</td><td rowspan="5">5</td><td>4.84</td><td>32.97</td><td rowspan="5">3</td><td rowspan="2">5.48</td><td rowspan="2">44.31</td><td rowspan="5">4</td><td rowspan="2">4.87</td><td rowspan="2">35.79</td><td rowspan="5">4</td><td rowspan="2">5.33</td><td rowspan="2">38.02</td></tr>
<tr><td>5.56</td><td>46.15</td></tr>
<tr><td rowspan="3">4.83</td><td rowspan="3">33.1</td><td>5.07</td><td>49.72</td><td rowspan="2">5.04</td><td rowspan="2">48.65</td><td>5.49</td><td>40.6</td><td>4.84</td><td>34.8</td></tr>
<tr><td>6.48</td><td>18.39</td><td>5.01</td><td>48.14</td><td>5</td><td>46.61</td></tr>
<tr><td>4.71</td><td>59.57</td><td>6.41</td><td>17.94</td><td>6.16</td><td>20.36</td><td>6.14</td><td>18.37</td></tr>
</table>

Figure S1 : Diagram of [Fe(PM-BiA)$_2$(NCS)$_2$]: **(a) Bia-P$_{mono}$** and **(b) Bia-P$_{ortho}$**, showing the labeling of the atoms. Thermal ellipsoids are drawn at 50% probability level.

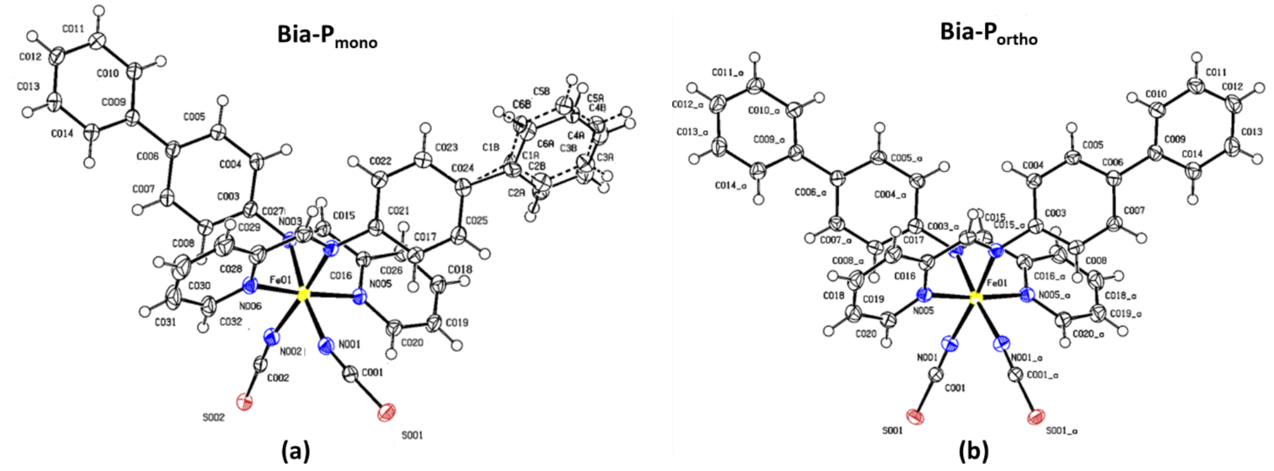

Figure S2 : Rietveld refinement of the powder diffraction pattern for **(a) Bia-P$_{mono}$** measured at PSI at 270 K **(b) Bia-P$_{ortho}$** measured at ESRF at 250 K. The blue and green ticks correspond to the Bragg reflections originating from **Bia-P$_{ortho}$** and **Bia-P$_{mono}$**, respectively. Small impurity peaks in **(a)** correspond to the orthorhombic polymorph with a phase fraction of nearly 5%. The open red circles are observed data points and the solid black line corresponds to the Rietveld fit. The pink solid line represents the difference between observed and calculated patterns.

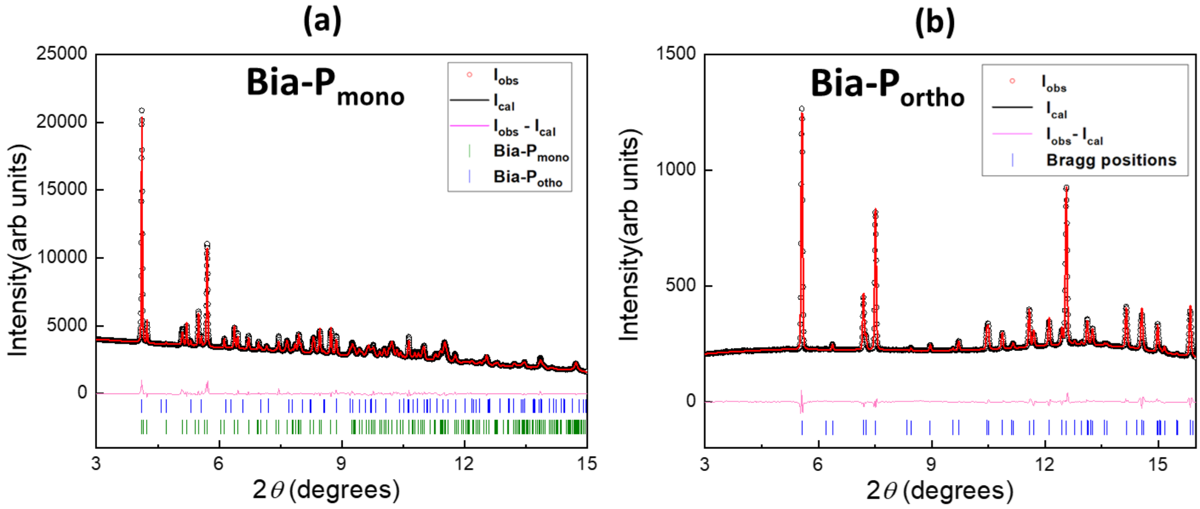

Figure S3 : Temperature evolution of the unit cell volume for the two polymorphs. The filled black square symbols black for **Bia-P$_{mono}$** and the filled red circles for **Bia-P$_{ortho}$** are from this study. Open stars are from ref [*a*]: (Buron-Le Cointe *et al.*, 2012). Lines are guides to the eyes. Error bars are the same size or smaller than the symbols.

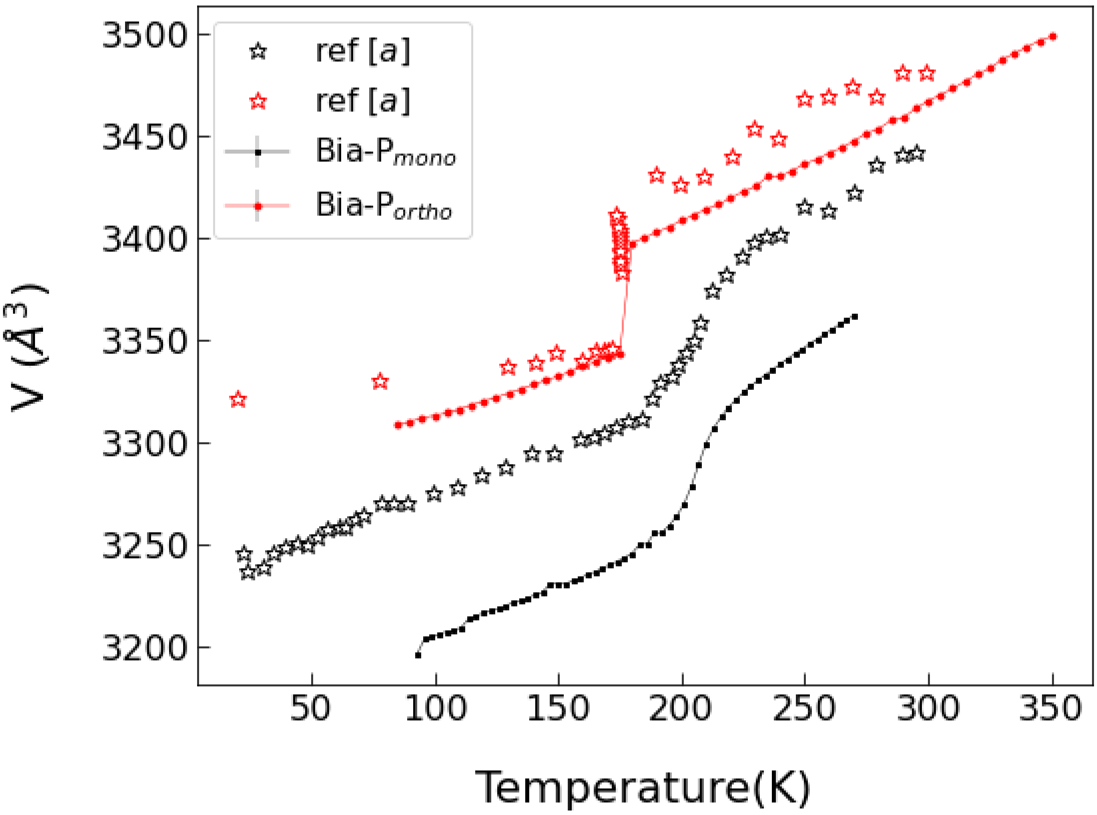

Figure S4 : Fe−N distances, Fe(U_{11}), and Fe(x) as a function of temperature for **Bia-P$_{ortho}$** for the second cooling **(a)** and heating **(b)** cycles across the SCO transition (169-190 K).

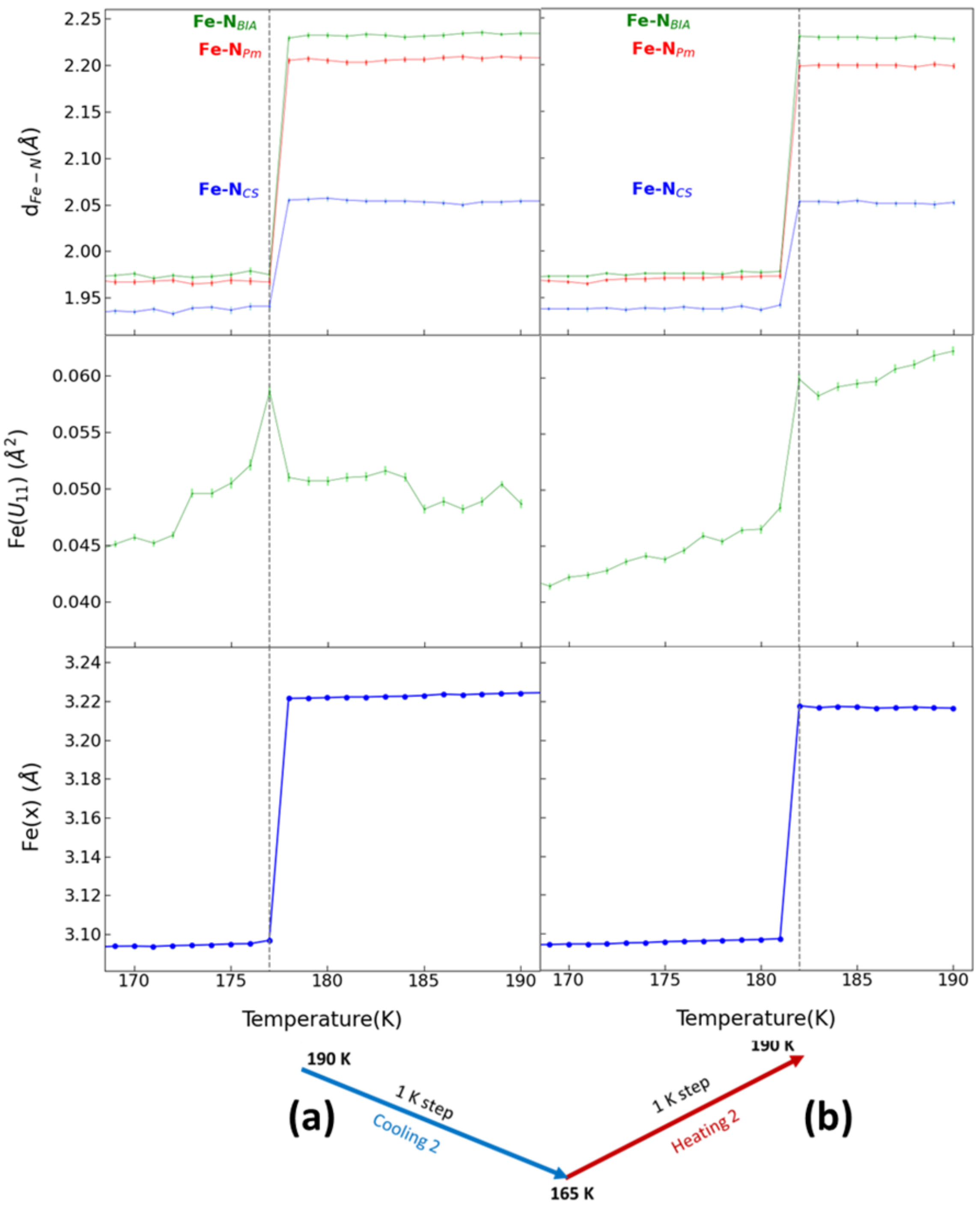

Figure S5 : (Top) The Fe−N−C(S) angle as a function of temperature. (Bottom). The NCS⁻ angle as a function of temperature. The red circles (**Bia-P$_{ortho}$**) and the full blue and open dark blue squares (**Bia-P$_{mono}$**) are from this study (the two colors are be used to differentiate the two NCS⁻ branches). Open blue and dark blue stars are from ref [*a*]:(Marchivie *et al.*, 2003). Red open star symbol are from ref [*b*]: (Létard *et al.*, 1998). The standard deviations are indicated by the shaded area. Lines are guides to the eyes.

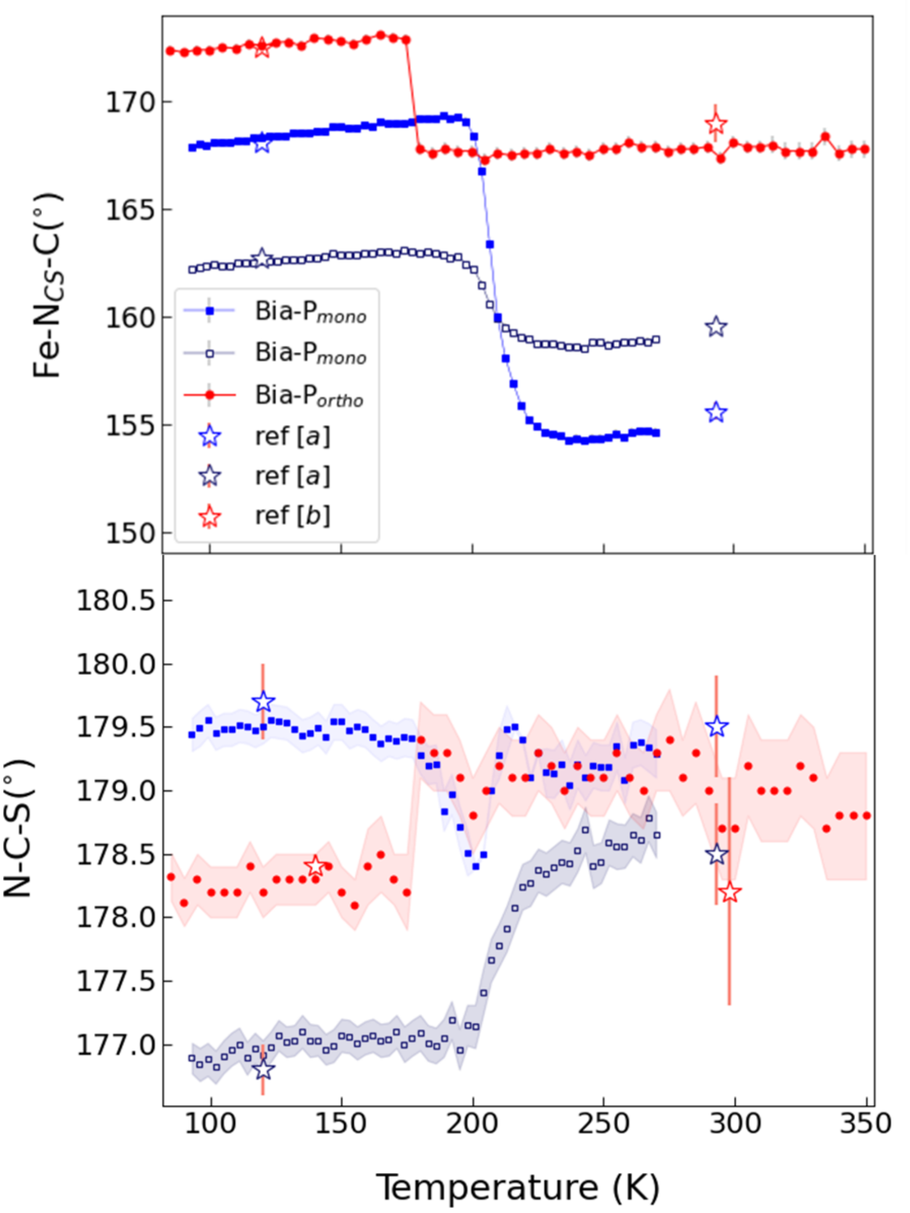

Figure S6 : Comparison of the evolution of the *a*-lattice parameter of **Bia-P$_{mono}$** with various parameters. Red dashed lines represented two temperature points (219 K and 183 K) and highlight the changes in trend. The gray shaded regions show the temperature range (243 K - 219 K), where part of the π-π interactions is changing their strength from moderate to strong. **(a)** Comparison to the high spin fraction estimated from the magnetization measurement **(b)** Comparison to the torsion angle between two biphenyl rings. **(c)** Comparison to the van der Waals interaction between two carbon atoms. **(d)** Comparison to the angle between the Fe−N−C in the thiocyanate arm.

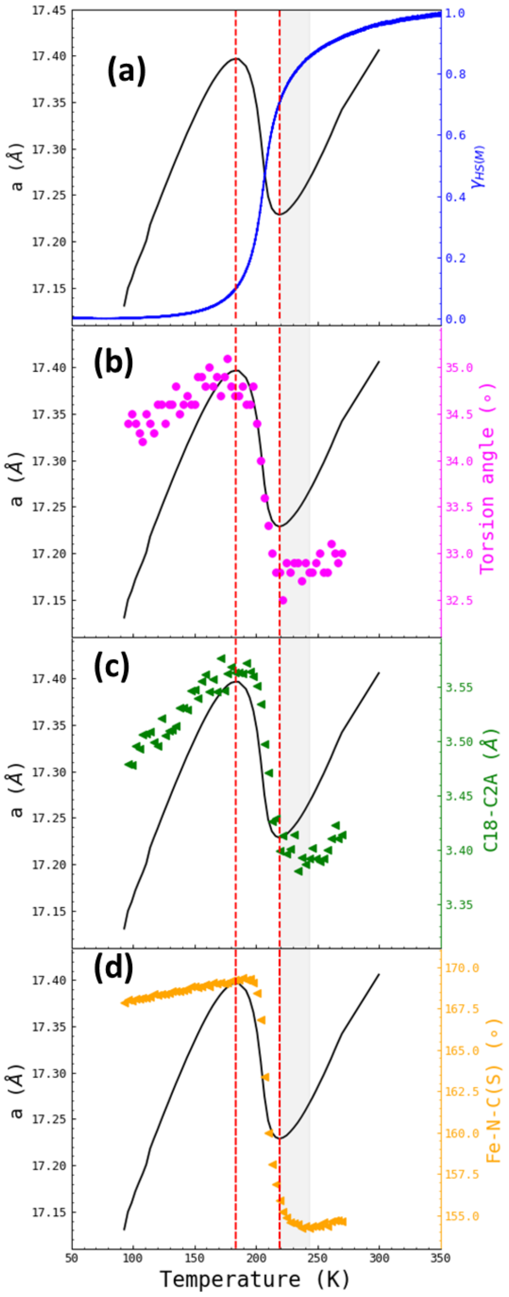

Figure S7 : (Right) The N_{Bia}−Fe−N_{NCS} angle as function of temperature; full red circles (**Bia-P$_{ortho}$**: N1−Fe−N3) and full blue and open dark blue squares (**Bia-P$_{mono}$**: N1−Fe−N3, N2−Fe−N4, respectively) are from this study. (Left) the N_{Pm}−Fe−N_{Pm} angle a function of temperature; full red circles (**Bia-P$_{ortho}$:** N5−Fe−N5) and full black squares (**Bia-P$_{mono}$** N5−Fe−N6) are from this study. Blue, dark blue, and black open stars are from ref [*a*]: (Marchivie *et al.*, 2003). Lines are guides to the eyes. Error bars are the same size or smaller than the symbols.

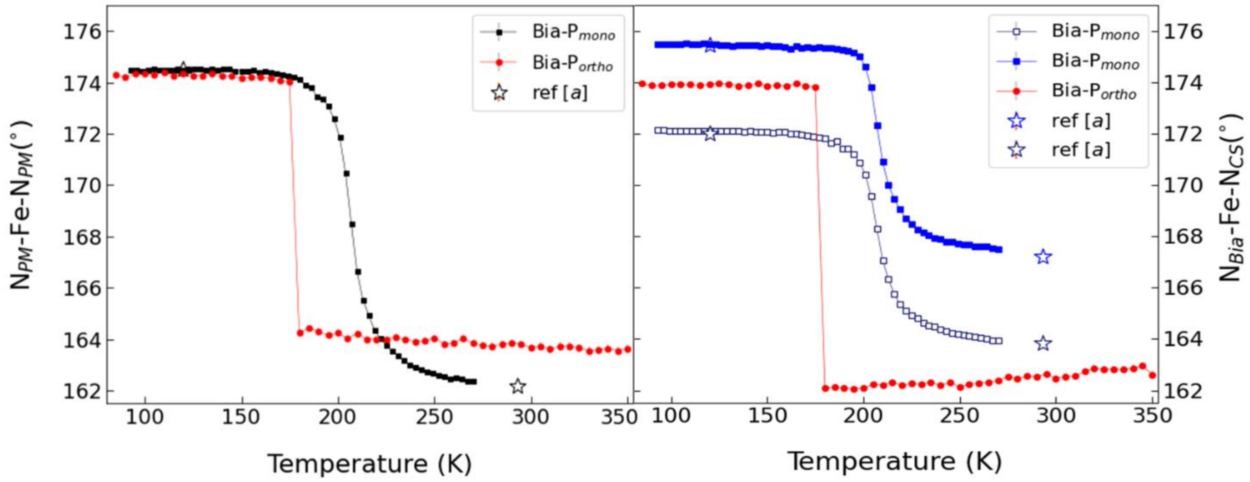

Figure S8 : (Right) The N_{Bia}−Fe−N_{NCS} angle as function of temperature; red circle (**Bia-P$_{ortho}$**: N1−Fe−N3) and full blue and open dark blue squares (**Bia-P$_{mono}$**: N1−Fe−N4, N2−Fe−N3, respectively) are from this study. (Left) the N_{Bia}−Fe−N_{Bia} angle a function of temperature; red circles (**Bia-P$_{ortho}$:** N3−Fe−N3) and full black squares (**Bia-P$_{mono}$** N3−Fe−N4) are from this study. Blue, dark blue, and black open stars are from ref [*a*]: (Marchivie *et al.*, 2003). Lines are guides to the eyes.

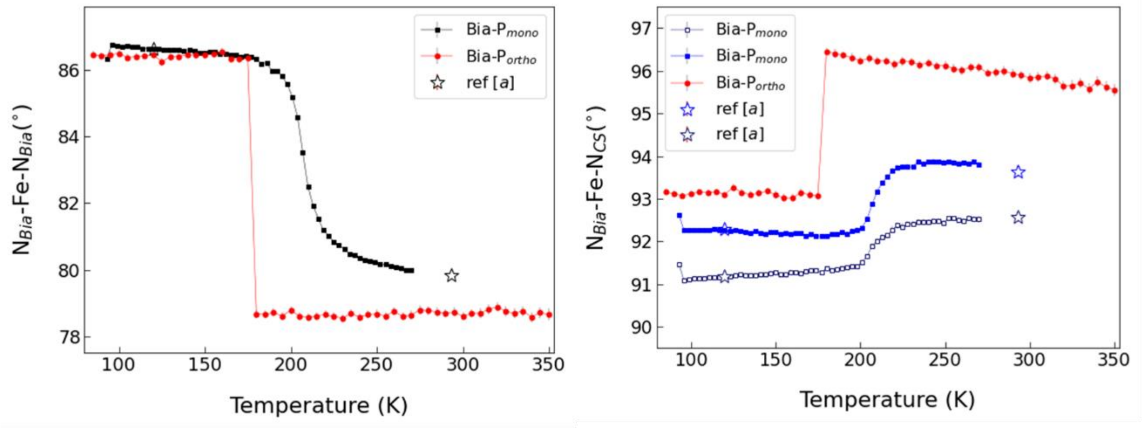

Figure S9 : The N_{PM}−Fe−N_{NCS} angle as function of temperature; red circles (**Bia-P$_{ortho}$**: N1−Fe−N5) and full blue and open dark blue squares (**Bia-P$_{mono}$**: N1−Fe−N3, N2−Fe−N6, respectively) are from this study. Blue and dark blue open stars are from ref [*a*]:(Marchivie *et al.*, 2003). Lines are guides to the eyes.

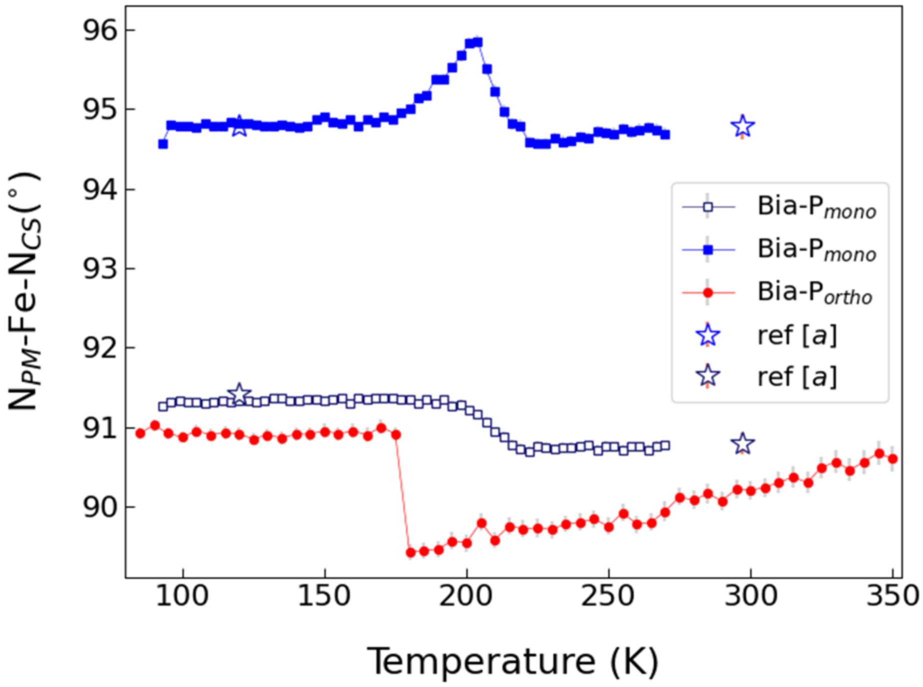

Figure S10: (Top) Σ: Angular distortion parameter of the 12 N−Fe−N bond angles. (Middle) ζ: Bond length distortion parameter. (Bottom) Θ: the deviation from ideal octahedral towards a trigonal prismatic coordination. Red circles (**Bia-P$_{ortho}$**) and black squares (**Bia-P$_{mono}$**) are from this study. Red and black open stars are from ref [*a*]:(Buron-Le Cointe *et al.*, 2012); open red circles and open black squares are from ref [*b*]:(Marchivie *et al.*, 2003); open red and black diamonds are from ref [*c*]:(Marchivie *et al.*, 2005). Lines are guides to the eyes. Error bars are the same size or smaller than the symbols.

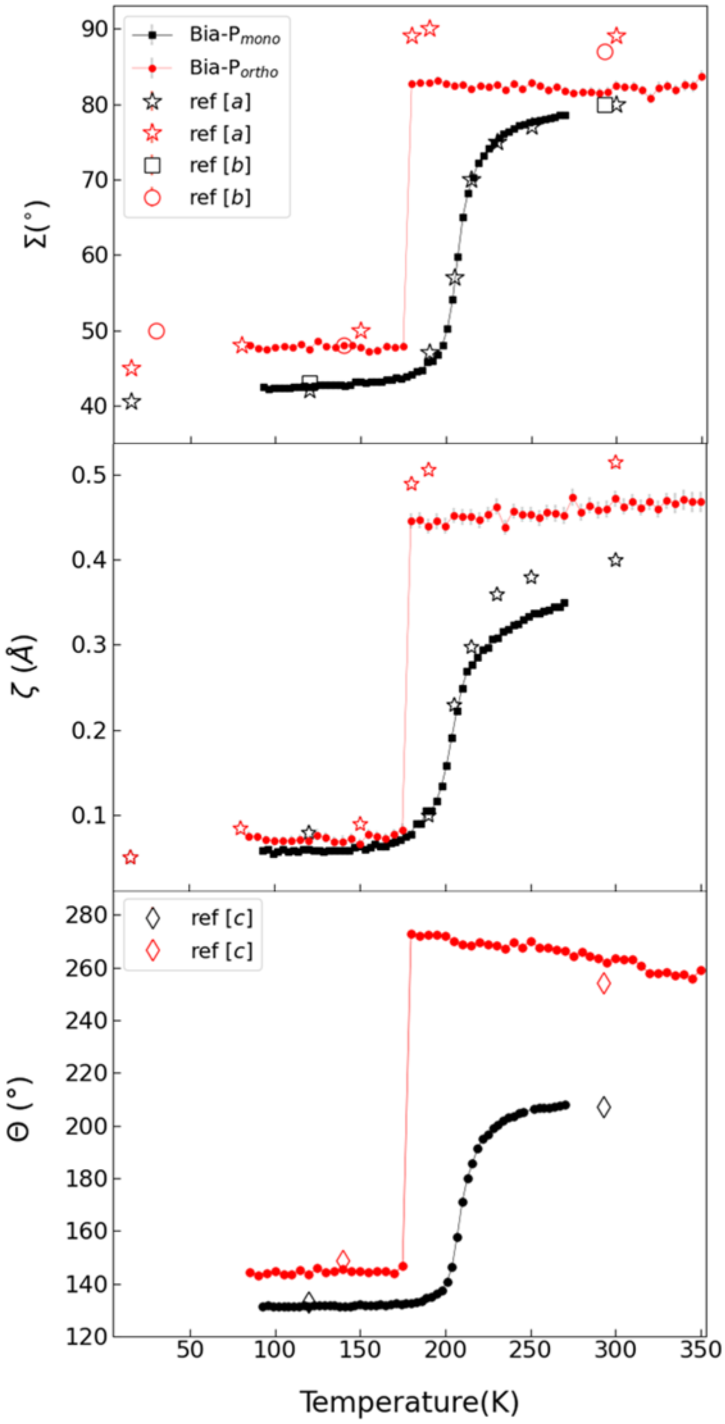

Figure S11: The evolution of the C−C bond length in the pyridine ring as a function of temperature. (Left) **Bia-P$_{mono}$**, (right) **Bia-P$_{ortho}$**. Lines are guides to the eyes.

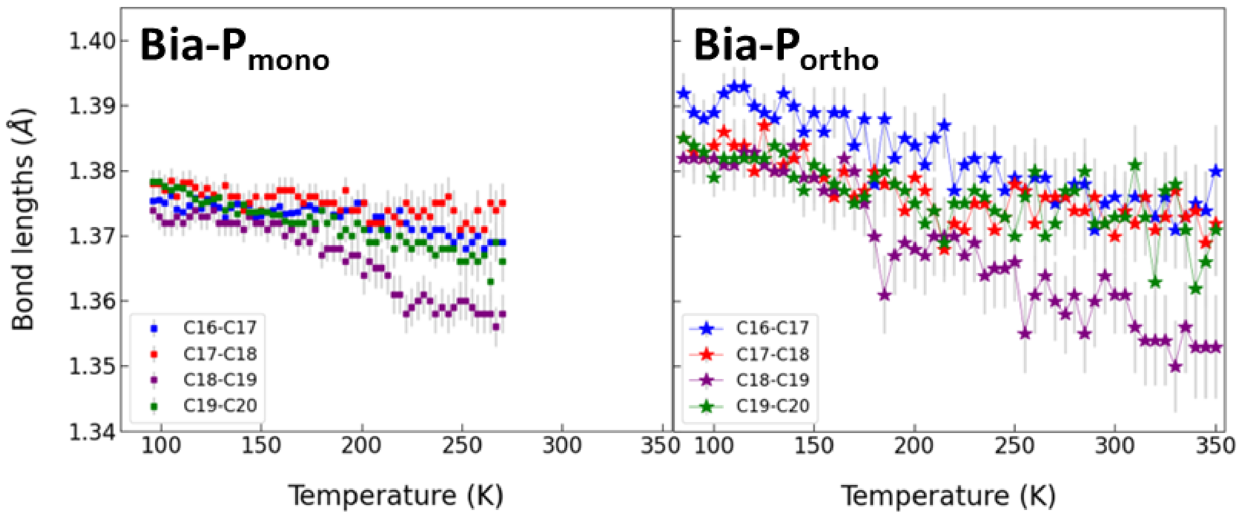

Figure S12: The evolution of the C−C bond length in the phenylene rings as a function of temperature. (left) **Bia-P$_{mono}$**, (right) **Bia-P$_{ortho}$**. Lines are guides to the eyes.

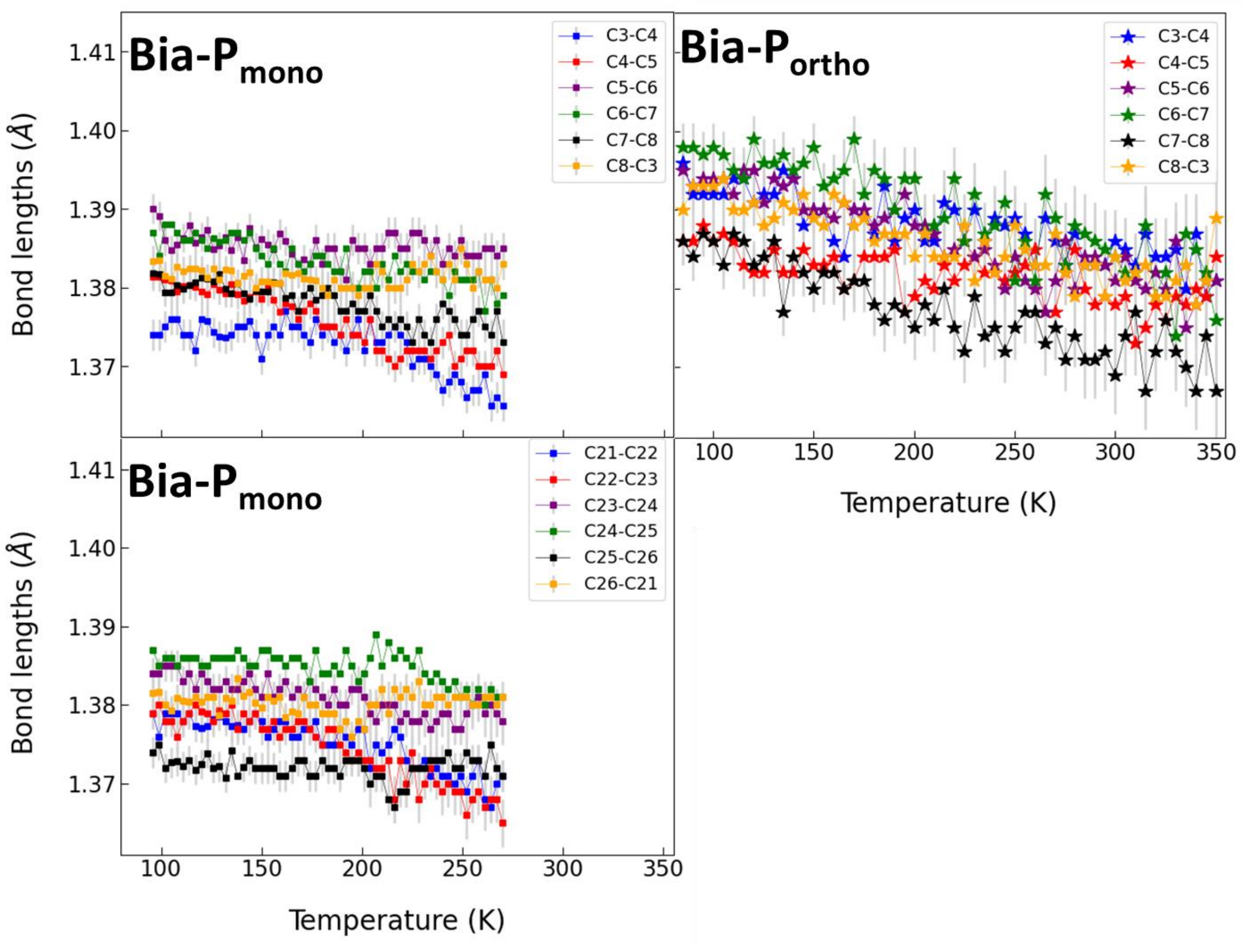

Figure S13: 3D packing of **Bia-P$_{\mathbf{mono}}$** showing the shortest contacts between phenyl rings. The strength depends on the distance between two centroids and the angle between the normal vectors of the two centroids. The interaction between the inner phenyl rings (centroid 1 and centroid 5) shows a strong interaction at all temperature points; however, centroid 7 & centroid 1 and, centroid 3 & centroid 5 exhibit moderate strengths in the HS state. At certain temperature points, they become strong, and in the LS state, they restore their moderate strength.

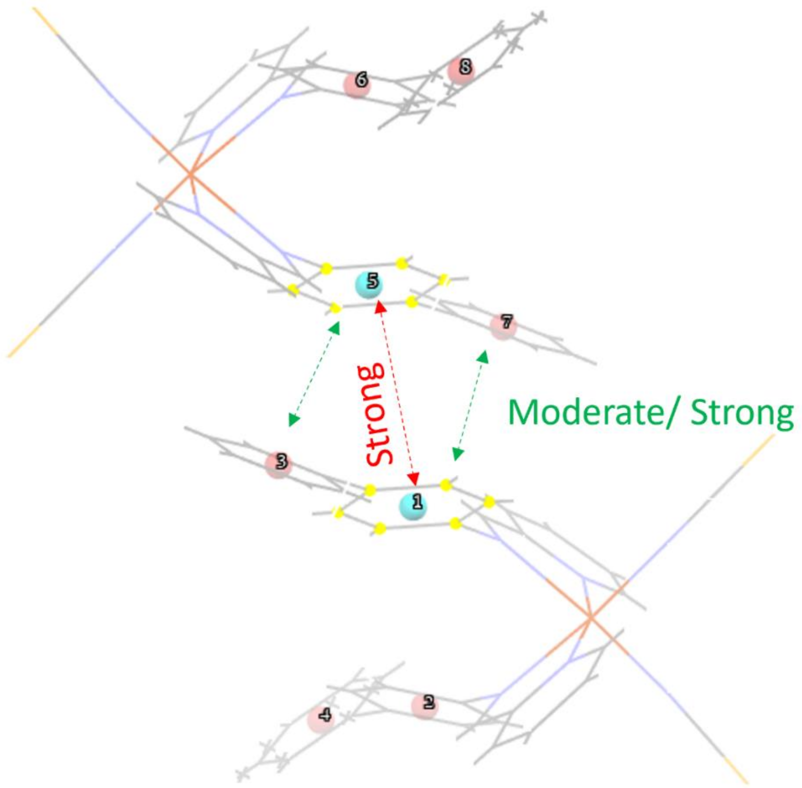

Figure S14: The N−Fe−N angle (N corresponds to NSC^- group) as a function of temperature. Red circles correspond to **Bia-P$_{\mathbf{ortho}}$** and black squares correspond to **Bia-P$_{\mathbf{mono}}$**. Filled symbols are obtained from this study. Open stars symbols are obtained from ref [*a*]:(Marchivie *et al.*, 2003). Lines are guides to the eyes. Error bars are the same size or smaller than the symbols.

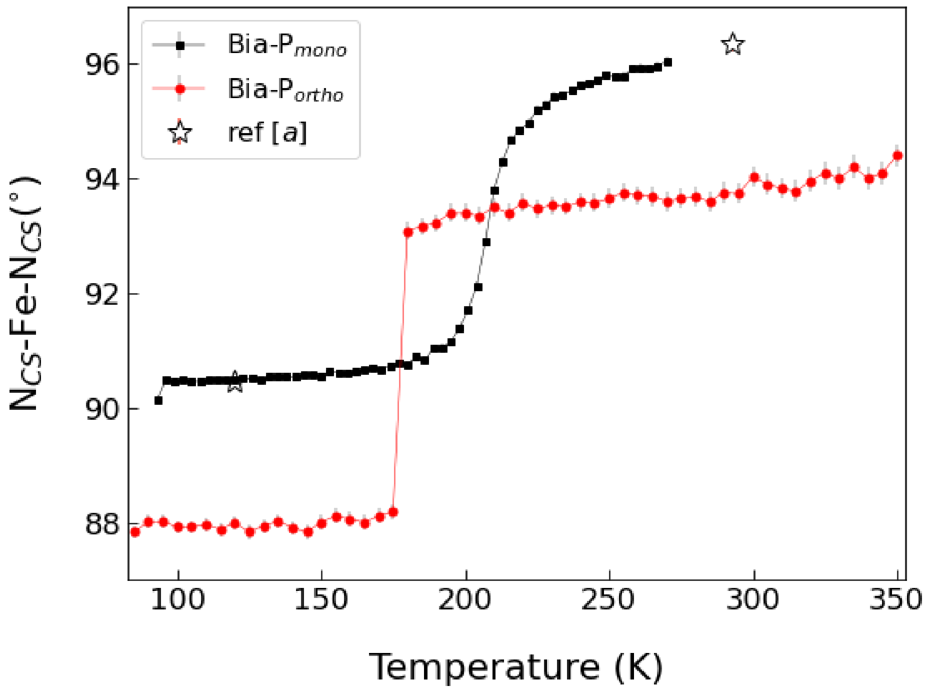

Figure S15: (Right) The C···C intermolecular shortest distance as a function of temperature in **Bia-P$_{ortho}$** exist only in the LS state. Contacts are considered short if C···C < 3.5 Å. $C_{11}\cdots C_1$ and $C_{14}\cdots C_{14}$ are the shortest contact in the intersheet layers. $C_{19}\cdots C_{10}$ is the shortest contact in the intrasheet layers. (Left) The shortest C···C intermolecular distances as a function of temperature in **Bia-P$_{mono}$**. Contacts are considered short if C···C < 3.5 A˚. $C_{27}\cdots C_{6A}$ and $C_{15}\cdots C_{11}$ are the shortest contact in the intersheet layers. $C_{31}\cdots C_{6A,}$ $C_{2A}\cdots C_{14,}$ $C_{3A}\cdots C_{29,}$ $C_{19}\cdots C_{10}$ are shortest contact in the intrasheet layers. $C_{2A}\cdots C_{14}$ shortest contact in the intrasheet layers shows a similar change in its trend as the *a* lattice parameter with decreasing the temperature.

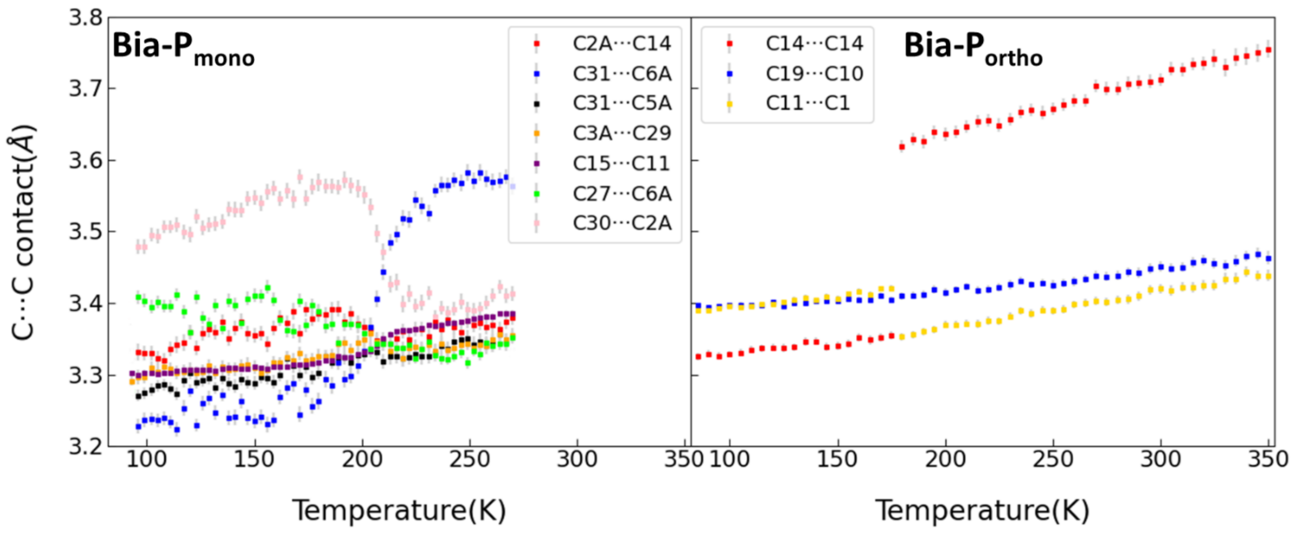

Figure S16: The mosaicity as a function of temperature for **Bia-P$_{ortho}$** during thermal cyclic measurements. The e1, e2, and e3 values correspond to crystal mosaicity and are obtained from CrysAlisPro software.

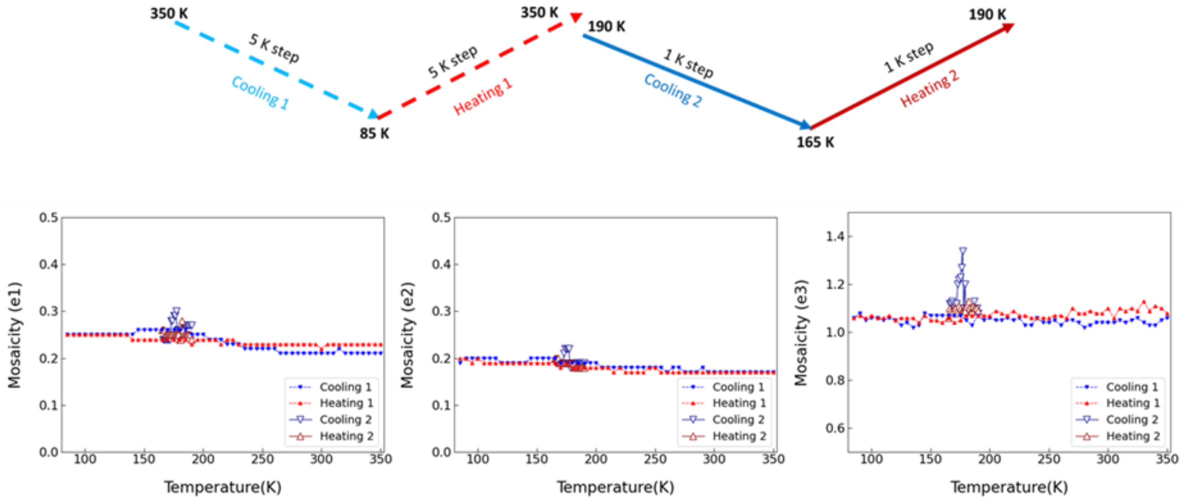

Figure S17 (a) Evolution of shortest Fe···Fe distance as a function of temperature for both polymorphs. **(b)** Illustration of the Fe-Fe distances in **Bia-P$_{ortho}$** **(c)** Fe···Fe distances as a function of the temperature of **Bia-P$_{ortho}$**, open stars are from ref [*a*]:(Létard *et al.*, 1998). Lines are guides to the eyes. Error bars are the same size or smaller than the symbols.

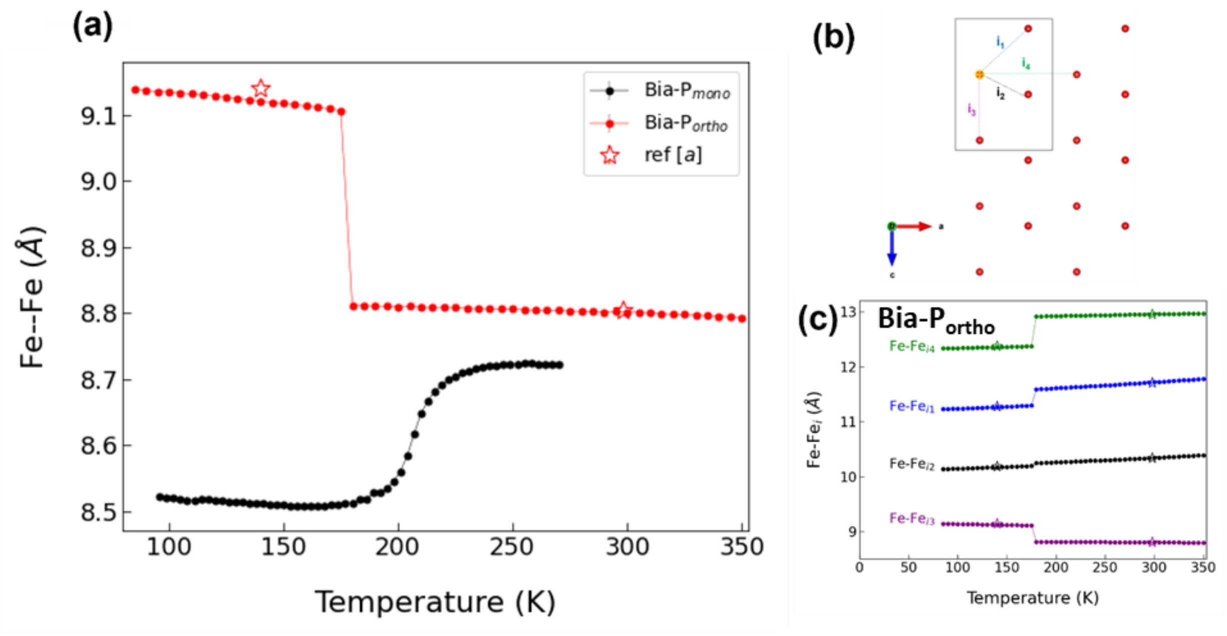

Supplement: Supplementary file 6 [file b-79-00354-sup6.pdf]
